# 3D Printed Sodiophilic Reduced Graphene Oxide/Diamane Microlattice Aerogel for Enhanced Sodium Metal Battery Anodes

**DOI:** 10.1002/advs.202417638

**Published:** 2025-03-31

**Authors:** Mengmeng Liu, Dezhi Kong, Ningning Chu, Gang Zhi, Hui Wang, Tingting Xu, Xinchang Wang, Xinjian Li, Zhuangfei Zhang, Hui Ying Yang, Ye Wang

**Affiliations:** ^1^ Key Laboratory of Material Physics Ministry of Education, School of Physics Zhengzhou University Zhengzhou 450052 China; ^2^ Pillar of Engineering Product Development Singapore University of Technology and Design 8 Somapah Road Singapore 487372 Singapore

**Keywords:** 3D printing, dendrite‐free morphology, diamane, sodiophilicity, sodium metal anode

## Abstract

Sodium metal anode holds great potential for high energy density sodium batteries. However, its practical utilization is impeded by significant volume change and uncontrolled dendrite growth. To tackle these issues, a three‐dimensional (3D) hierarchical porous sodiophilic reduced graphene oxide/diamane (rGO/diamane) microlattice aerogel is constructed by a direct ink writing (DIW) 3D printing (3DP) method. The molten Na is diffused into the rGO/diamane host to form Na@rGO/diamane anode, which can deliver an ultra‐high capacity of 78.60 mAh cm^−2^ (1090.94 mAh g^−1^). Benefiting from uniform ion distribution and homogeneously distributed sodiophilic diamane enabled dendrite‐free deposition morphology, the Na@rGO/diamane anodes exhibit a long cycle‐life of over 7200 h at 1 mA cm^−2^ with 1 mAh cm^−2^. Furthermore, the Na@rGO/diamane anode also enhances the long‐term stability at an elevated operation temperature of 60 °C, sustaining a prolonged lifespan of 400 h at 1 mA cm^−2^ with 1 mAh cm^−2^. Notably, when integrated with the Na_3_V_2_(PO_4_)_3_@carbon (NVP@C) cathode and Na@rGO/diamane anode, the full cell delivers sustained longevity, maintaining a lifespan of over 2000 cycles with a capacity retention rate of 95.72%. This work sheds new insights into the application of diamane for the development of stable and high‐performance sodium metal batteries.

## Graphical abstract

A 3D printed sodiophilic rGO/diamane microlattice aerogel with uniform ions flux and exceptional sodiophilic features is demonstrated as a stable host for sodium metal anode. The sodiophilic diamane nanoflakes regulate the uniform deposition of metallic Na with a dendrite‐free deposition morphology, achieving an ultra‐high areal capacity of 78.60 mAh cm^−2^ and a cycle lifespan of 7200 h.

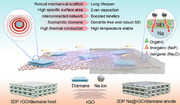

## 1 Introduction

Sodium metal, with ultra‐high theoretical specific capacity (1166 mAh g^−1^) and low redox potential (−2.71 V vs standard hydrogen electrode), is regarded as a promising anode material for sodium metal batteries (SMBs).^[^ ^1^, ^2^, ^3^ ^]^ Nevertheless, the practical application of SMBs has been hindered by significant volume change and uncontrolled growth of sodium dendrites.^[^ ^4^, ^5^, ^6^ ^]^ These problems are bound to trigger repeated breakage and rebuilding of the solid electrolyte interphases (SEI), poor Coulombic efficiency (CE), rapidly decaying capacity, and even serious safety issues.^[^ ^7^, ^8^, ^9^ ^]^ To tackle these issues, extensive efforts have been devoted to exploring the importance of mechanical properties by construction of artificial SEI layer (e.g., Na_2_Se/V,^[^ ^10^ ^]^ HCOONa,^[^ ^11^ ^]^ and Na‐alloy^[^ ^12^ ^]^) and solid state electrolyte (e.g., metal‐organic framework (MOF),^[^ ^13^ ^]^ amorphous NaTaCl_6_ halide^[^ ^14^ ^]^ and Na_5_SmSi_4_O_12_^[^ ^15^ ^]^), which can build a robust or high Young's modulus layer on or above the two‐dimensional (2D) sodium metal foil to efficiently restrain sodium dendrite growth. Since the sodium metal interface needs to move a long distance of ≈27 µm to provide a capacity of 3 mAh cm^−2^ for practical application according to the theoretical specific capacity and density of Na metal, it is a challenge to keep the interface integrity with high stability. Therefore, it is rational to design a three‐dimensional (3D) scaffold to spatially confine the active Na metal mechanically and minimize the volume change in the porous accommodation during repeated deposition/stripping processes.^[^ ^16^, ^17^, ^18^ ^]^ Moreover, the 3D scaffold host also provides a large surface area to reduce the current density and against the inhomogeneous sodium flux and deposition.^[^ ^19^, ^20^, ^21^, ^22^ ^]^

A superior 3D host for long‐cycle performance SMBs should have the merits of superior mechanical strength to restrain the uneven Na deposition and maintain the structural integrity, the light weight to maximize the specific capacity, the high electrochemical stability against the Na metal and the electrolyte environment, the appropriate surface area with porous structure to accommodate the Na metal and a sodiophilic interface to guide the sodium ion migration and deposition.^[^ ^16^, ^18^, ^20^, ^23^ ^]^ It is essential and critical to select a suitable sodiophilic material to achieve dendrite‐free morphology with long‐cycle stability SMBs. Diamane, as an emerging 2D nanodiamond, possesses high Young's modulus and chemical inertness which can give 3D host the ability to respond to volume change and avoid side effects.^[^ ^24^ ^]^ Moreover, the abundant oxygen‐containing functional groups on the surface of diamane result in sodiophilicity, which can guide the uniform deposition of sodium ions to inhibit dendrite formation.^[^ ^16^, ^25^, ^26^ ^]^ In short, the physical, chemical, and electrochemical properties of diamane are suitable for constructing a sodiophilic interface for dendrite‐free deposition morphology.^[^ ^27^ ^]^

**Figure 1:**
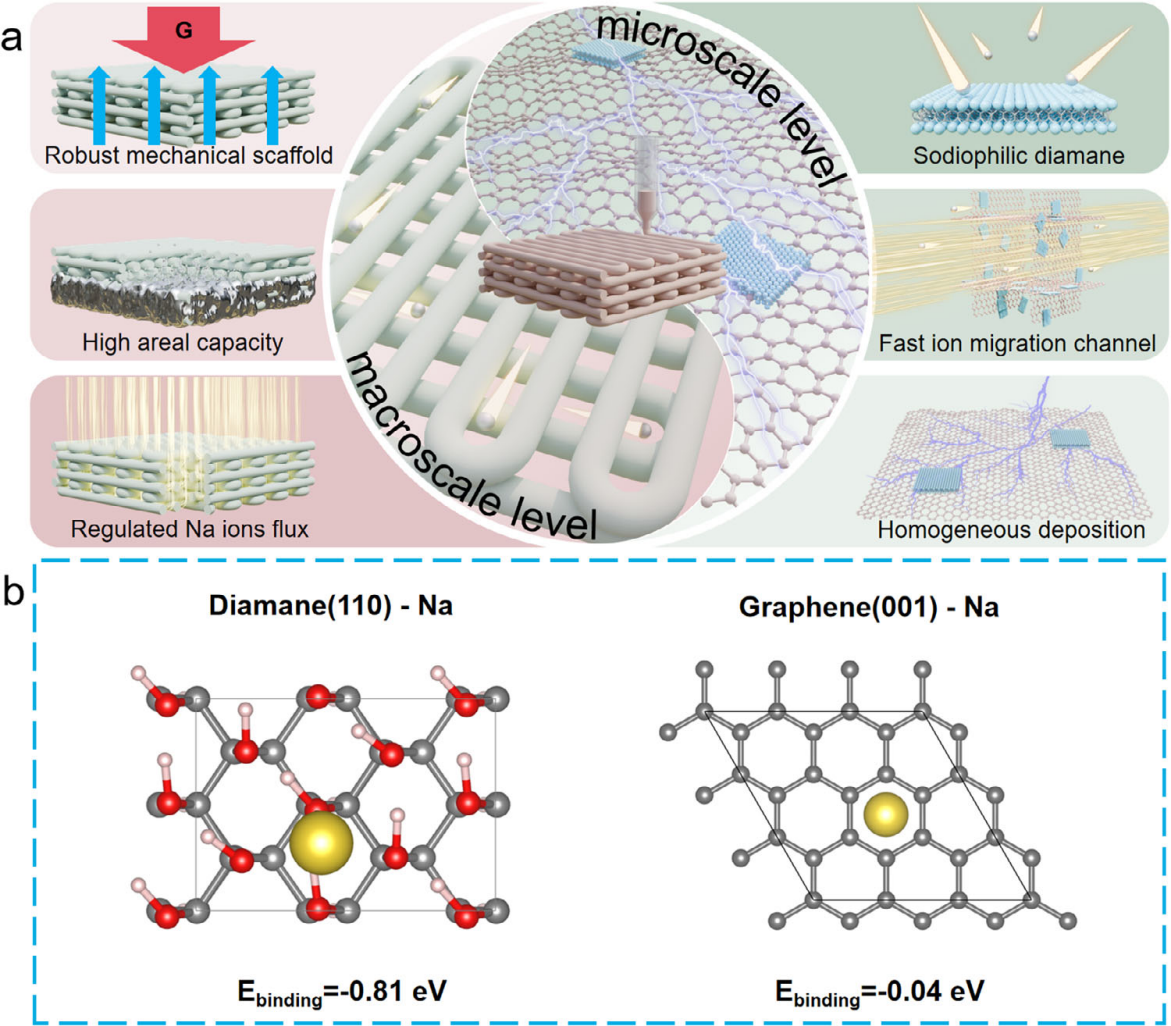
a) Schematic illustration of 3D printed rGO/diamane microlattice electrode with a hierarchical porous frame serves as sodium metal anode host. b) Binding energies of diamane‐Na and graphene‐Na calculated by DFT simulation.

Herein, a hierarchical porous microlattice aerogel electrode composed of reduced graphene oxide/diamane (rGO/diamane) was fabricated by a direct ink writing (DIW) 3D printing (3DP) technology. The electrode fabricated by 3D printing technology offers advantages of enhanced areal capacity and energy density mainly due to the large active material mass loading, thick electrode, hierarchical porous structure, and fast ion transportation.^[^ ^28^ ^]^ Therefore, it is meaningful to explore this advanced technology for high‐performance electrode fabrication. To practically scale up the 3D printing process, the throughput can be improved and the cost can be reduced by using multiple nozzles. From the macroscale level, the artificial hierarchical microlattice is employed to provide a robust mechanical scaffold to accommodate the sodium metal with an ultra‐high areal capacity (78.60 mAh cm^−2^). RGO is used to construct a microlattice scaffold to support the whole electrode, and the diamane is used to construct a sodiophilic interface. Furthermore, the 3D printed rGO/diamane (3DP rGO/diamane) electrode with pores arranged periodically in orthometric directions can regulate the sodium ion flux.^[^ ^19^, ^20^ ^]^ The well‐designed structure can constrain the volume change inside the electrode pores during repeated deposition/stripping processes, thus inhibiting the excessive growth of dendrites.^[^ ^29^, ^30^ ^]^ From the microscale level, the interwoven rGO sheets not only increase the specific surface area for reduced local current density but also form a continuous electron transfer and ion transportation path which promotes ions/electrons transport kinetics.^[^ ^20^, ^22^ ^]^ More importantly, the diamane nanoflakes which are homogeneously distributed on rGO sheets have strong sodiophilic properties and can induce transverse migration of Na ions with a more uniform flux, leading to a dendrite‐free morphology (**Figure** **1**a).^[^ ^26^, ^31^ ^]^ The sodiophilic property of diamane was proved by first‐principles density functional theory (DFT) calculation (Figure 1b). The binding energy between diamane and Na is −0.81 eV, which is stronger than that between graphene and Na (−0.04 eV), indicating that the Na ion prefers to nucleate and deposit on diamane. With the above merits, the resultant 3DP rGO/diamane electrode exhibits an ultra‐high CE of 99.95% at 2 mA cm^−2^ with 1 mAh cm^−2^ for 2000 h. The symmetric Na@rGO/diamane anode delivers a long cycling lifespan of more than 7200 h at 1 mA cm^−2^ with 1 mAh cm^−2^. Furthermore, a full cell composed of the 3DP Na_3_V_2_(PO_4_)_3_@C‐rGO (NVP@C‐rGO) cathode and 3DP Na@rGO/diamane anode delivers a long lifespan of over 2000 cycles with a high initial specific capacity of 95.75 mAh g^−1^ and a low capacity decay rate of 0.0027% per cycle at 1 C.

## 2 Results and Discussion

### 2.1 Preparation and Characterization of 3DP Na@rGO/Diamane Electrodes

**Figure 2:**
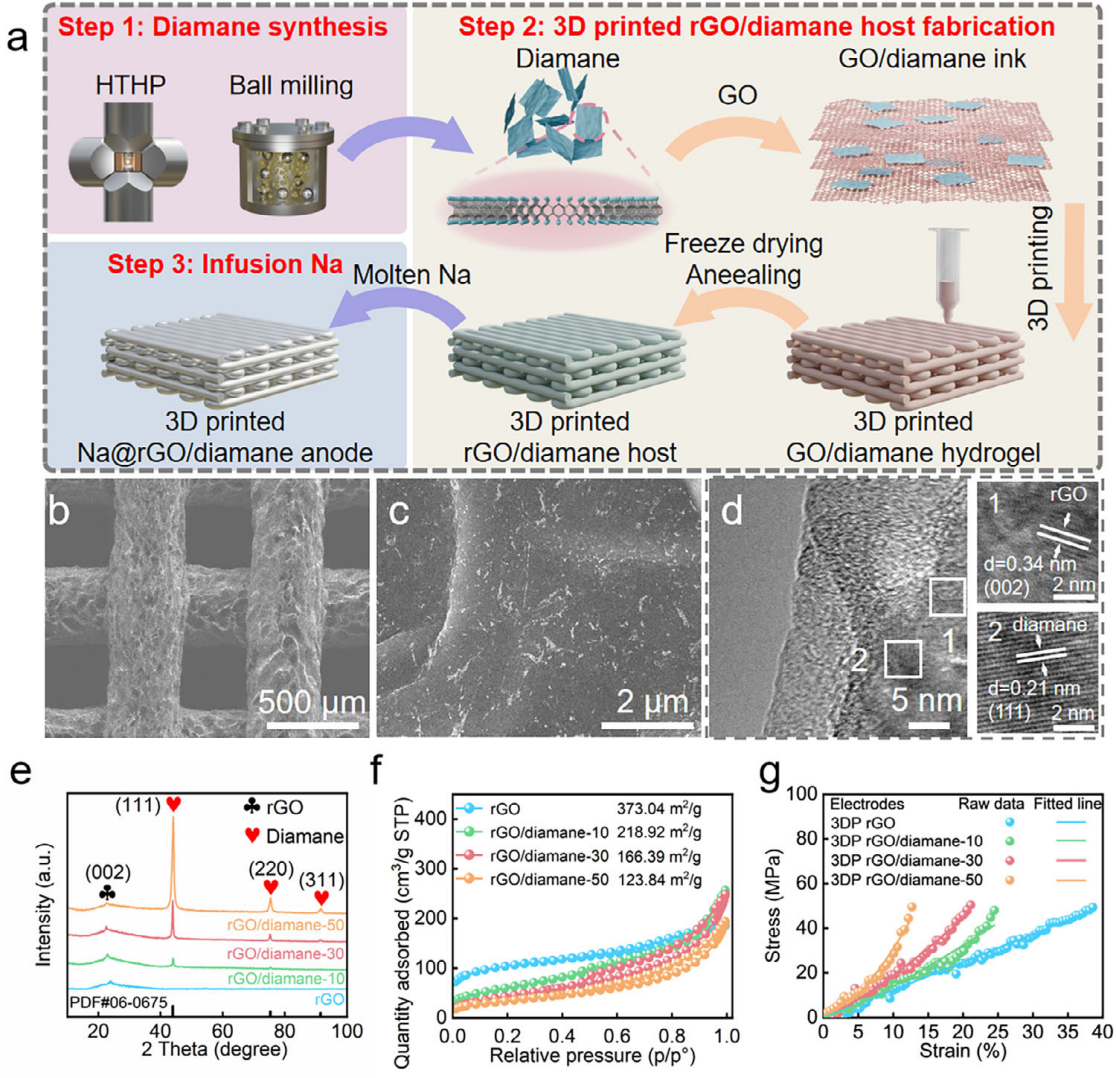
a) Schematic diagram of the synthesis processes of the 3DP Na@rGO/diamane anode. b,c) SEM and d) TEM images of 3DP rGO/diamane‐30 microlattice aerogel. e) XRD patterns, f) N_2_ adsorption/desorption isotherms, and g) reduced storage modulus of 3DP rGO, rGO/diamane‐10, 30, and 50 electrodes.

The fabrication of the 3DP Na@rGO/diamane anode can be divided into three steps: diamane synthesis, 3D printed rGO/diamane host fabrication, and infusion of Na (**Figure** **2**a). Initially, diamane nanoflakes were mechanically exfoliated from micro‐sized diamonds synthesized by high‐temperature high‐pressure (HTHP) process using a mechanical ball milling technique. The diamane nanoflake, which has a 2D structure morphology (Figure S1, Supporting Information), shows a typical lateral size of ≈400 nm with a thickness of 4.55 nm (Figure S2 and S3, Supporting Information).^[^ ^31^ ^]^ The X‐ray diffraction (XRD) shows the peaks centered at 43.8, 75.3, and 91.5° belonging to (111), (220), and (311) crystal planes of diamane (PDF#06‐0675), respectively (Figure S4, Supporting Information).^[^ ^32^ ^]^ These results demonstrate that the synthesized diamane still retains the diamond crystal structure without any additional phase generated during the exfoliation and multiple acid treatment processes. Further Raman measurement identifies that there is only one peak at ≈1332 cm^−1^ corresponding to sp^3^ carbon (sp^3^‐C) (Figure S5, Supporting Information), without any additional sp^2^ carbon signal, which is different from the conventional nanodiamond.^[^ ^31^ ^]^ This result indicates that the surface of diamane is physically and chemically stable. As a 2D material, diamane also has a large specific surface area of 204.70 m^2^ g^−1^ (Figure S6, Supporting Information). The surface functional groups of diamane are characterized by the Fourier‐transform infrared spectroscopy (FTIR). The peaks of the O─H bond are observed at 3450 and 1615 cm^−1^, and the two other peaks at 1754 and 1120 cm^−1^ correspond to the stretching vibration bonds of C═O and ─COOH, respectively (Figure S7, Supporting Information).^[^ ^33^ ^]^ X‐ray photoelectron spectroscopy (XPS) further affirms that oxygen‐containing groups appear on the surface of diamane, the corresponding peaks are for C─O (532.4 eV), C═O (530.9 eV) and O─H (529.5 eV) (Figure S8, Supporting Information).^[^ ^25^ ^]^ Consequently, the synthesized diamane is distributed with oxygen‐containing functional groups (Figure S9, Supporting Information). The abundance of these oxygen‐containing functional groups on the diamane surface contributes to its sodiophilicity.

The second step is the fabrication of the 3D printed rGO/diamane electrode. Since the diamane is endowed with functional groups such as O─H, ─COOH, and C═O, it is easily mixed with the graphene oxide (GO) solution without forming aggregation. The function of GO is used to prepare the 3D printing ink with appropriate viscosity and rheological properties. The 3D printable ink was formulated through mixing and centrifugation to remove excess water, yielding a gelatinous slurry with appropriate viscosity and rheological properties for 3D printing. Thereafter, the prepared ink was extruded into desired patterns and dimensions for electrodes using DIW technology. Subsequently, the printed GO/diamane electrode was subjected to freeze‐drying to preserve its microlattice architecture and remove the excess water, creating plenty of microcavities. This was followed by annealing to reduce the GO/diamane into rGO/diamane microlattice, which enhances electrical conductivity and improves mechanical strength. The composition of diamane within the 3DP rGO/diamane microlattice aerogel varies with different proportions (10%, 30%, and 50%) relative to the total mass of diamane and GO, denoted as 3DP rGO/diamane‐10, 30, and 50, respectively. It is worth mentioning that the 3D printing technique offers the flexibility to create diverse patterns with varying thicknesses (Figure S10a,b, Supporting Information). Additionally, the 3D printed rGO/diamane microlattice aerogel exemplifies the advantage of being ultra‐lightweight, weighing ≈3–4 mg, as demonstrated by a 3DP rGO/diamane electrode suspended by a dandelion flower without shape deformation (Figure S10c, Supporting Information). In this work, a 3‐layer (one vertical and one horizontal layer counted as one layer) 3DP rGO/diamane electrode was fabricated with a thickness of 2 mm and size of 1 cm × 1 cm (Figure S11, Supporting Information).

The 3DP rGO/diamane‐30 microlattice aerogel consists of regularly arranged filaments (average diameter: ≈400 µm) and periodic submillimeter channels (≈350 µm) as shown in Figure 2b. These filaments are composed of rGO sheets with a uniform dispersion of diamane nanoflakes and porous microcavities (Figure 2b,c). The 3DP filament features an extensive network of micropores and mesoporous, which are created by the interconnected rGO sheets. The interconnected filaments form a robust framework, attributed to π‐π stacking.^[^ ^29^ ^]^ An enlarged image taken using transmission electron microscopy (TEM) displays the uniform distribution of diamane nanoflakes on the rGO surface and the lattice spacing of 0.21 and 0.34 nm correspond to the (111) crystal face of diamane and the (002) crystal face of rGO, respectively (Figure 2d).^[^ ^31^, ^34^ ^]^ The 3DP rGO, rGO/diamane‐10, and rGO/diamane‐50 microlattice aerogels exhibit similar morphologies, with interconnected filaments and channels, but different quantities of diamane nanoflakes on the rGO nanosheets (Figures S12–S14, Supporting Information).

The XRD patterns of 3DP rGO and rGO/diamane microlattice aerogels are shown in Figure 2e. The main peak of rGO at 26.2° is attributed to (002) crystal plane of graphene.^[^ ^35^, ^36^ ^]^ Moreover, there are three other main peaks at 43.8, 75.3, and 91.5° of rGO/diamane attributing to the (111), (220), and (311) crystal planes of diamane (PDF#06‐0675), respectively (Figure S4, Supporting Information).^[^ ^32^ ^]^ Furthermore, the surface areas of 3DP rGO, rGO/diamane‐10, 30 and 50 are 373.04, 218.92, 166.39, and 123.84 m^2^ g^−1^, respectively (Figure 2f). The reduction in surface area with increasing content of diamane may be attributed to the blocking of micropores and mesoporous by diamane, which consequently reduces the exposed specific surface area.^[^ ^30^ ^]^ It is interesting to note that the mechanical strength is increased with the increasing ratio of diamane. As shown in Figure 2g, under the same pressure, the electrode compression strain decreases with increasing diamane content. The 3DP rGO/diamane electrodes exhibit superior mechanical strength due to the high Young's modulus of diamane integrated with rGO sheets.^[^ ^27^ ^]^ The 3DP rGO/diamane electrodes have significant advantages in inhibiting the volume change and the growth of Na dendrites through high mechanical strength.^[^ ^17^ ^]^ In addition, 3DP rGO and rGO/diamane electrodes show better electrolyte wettability, as the contact angles of 3DP rGO (≈0°) and rGO/diamane (≈0°) are much smaller than that of Cu (31.3°) (Figure S15, Supporting Information).^[^ ^37^ ^]^

The third step of the fabrication of Na@rGO/diamane anode is infusing the molten Na into the rGO/diamane. Infusing Na into rGO/diamane offers advantages over electrodeposition, including a more uniform Na distribution, which enhances electrochemical performance by ensuring consistent Na availability. The infusion process is also less aggressive to preserve structural integrity. Additionally, infusion is simpler and more scalable without complex electrochemical setups. While electrodeposition provides precise localization and controlled morphology, infusion is better suited for achieving homogeneous Na incorporation and maintaining rGO/diamane's properties. In the Ar‐filled glovebox, once the rGO/diamane electrode contacted with the molten Na, Na metal was spontaneously diffused into the 3DP rGO/diamane aerogel and mitigated into the entire electrode within 5 s (Figure S16 and Video S1, Supporting Information), which is less than that into the 3DP rGO host (>30 s, Figure S17 and Video S2, Supporting Information). This phenomenon can be attributed to the high affinity between the molten Na and the rGO/diamane, coupled with the capillary action generated by the microcavities within the printed scaffold.^[^ ^38^ ^]^ After the molten Na was infused into the electrode, the mass of the electrode can reach 60 mg or more. From the SEM image shown in Figure S18 (Supporting Information), the Na@rGO/diamane electrode still retains a 3D porous architecture with the Na metal firmly attaching to the rGO nanoflakes. This phenomenon proves that rGO/diamane is sodiophilic to attract the molten Na metal.^[^ ^39^ ^]^

### 2.2 Electrochemical Performance Evaluation

Prior to the electrochemical performance evaluation of the 3DP Na@rGO/diamane anode, the electrochemical parameters of the host were measured, including CE and nucleation overpotential. CE measurement was carried out to gain insight into reversibility of Na metal deposition/stripping behavior on various hosts based on the half cell without molten Na diffusion into the electrodes. This half cell comprises the bare Na foil as the counter electrode and a working electrode (Cu foil, 3DP rGO or rGO/diamane electrodes). As shown in Figure S19 (Supporting Information), the CE curve of 3DP rGO/diamane‐30 electrode is stable with an average CE of 99.95% at 2 mA cm^−2^ with 1 mAh cm^−2^ over 2000 cycles. Similarly, the CEs of the 3DP rGO/diamane‐10 and 50 electrodes are fluctuated gently with the average CEs of 99.59% and 99.32% for 2000 cycles, respectively. In contrast, the average CE of 3DP rGO electrode is 99.08% fluctuated between 90% and 100% for ≈800 cycles and then short‐circuited. The 2D planar Cu electrode shows the worst CE of 98.33% fluctuating wildly between 70% and 100% for ≈250 cycles and then short‐circuited. This is due to the formation of an unstable SEI layer and Na dendrites.^[^ ^40^ ^]^ This is proven by the ex‐situ SEM investigation which was performed to analyze morphology of Cu, 3DP rGO, and 3DP rGO/diamane electrodes after 10 cycles. As shown in Figure S20 (Supporting Information), the surface of the cycled Cu electrode has irregular bumps and pits due to the uneven deposition of Na metal. In addition, the huge volume change of the Cu electrode can lead to repeated breakage of the SEI. For the 3DP rGO electrode, the surface retains its original morphology but there is still a small amount of “dead Na”. In contrast, the surface of the 3DP rGO/diamane electrode still maintains a layered structure without any dendrites or “dead Na”. Additionally, compared to the 3DP rGO electrode, Na deposition on the 3DP rGO/diamane electrode is deposited uniformly throughout the 3D electrode through the EDS elemental mapping of the cross‐sectional images, rather than concentrated at the top of the 3D electrode due to the “top‐growth effect”.^[^ ^41^ ^]^

The rate performance of Cu, 3DP rGO, and rGO/diamane‐10, 30, and 50 electrodes was evaluated at a fixed areal capacity of 2 mAh cm^−2^ at various current densities from 0.5 to 5 mA cm^−2^ (Figure S21, Supporting Information). It can be seen that the voltage profile of the 3DP rGO/diamante‐30 electrode at various current densities sustains a stable curve with a sharp initial drop to show the nucleation, followed by a slow rise to a smooth plateau for Na plating. The Na nucleation overpotentials of Cu, 3DP rGO, and rGO/diamane‐10, 30, and 50 electrodes at various current densities are revealed in Figure S22 (Supporting Information). The nucleation overpotentials of the 3DP rGO/diamane‐30 electrode are 3.9, 7.5, 15.5, 20.4, and 24.9 mV at current densities of 0.5, 1, 2, 4, and 5 mA cm^−2^, which are the lowest overpotential at each current density among all measured electrodes. It is no doubt that the overpotential is reduced with the increase in the diamane ratio due to the sodiophilicity of diamane. It is worth noting that the nucleation overpotential of 3DP rGO/diamane‐50 electrode is higher than that of 3DP rGO/diamane‐30 electrode. The possible reason is the reduced conductivity of the non‐conductive diamane (Figure S23, Supporting Information) and the reduced specific surface area.^[^ ^30^ ^]^ This phenomenon was also observed in other studies.^[^ ^16^, ^20^ ^]^ The reduction in nucleation overpotential is mainly due to the sodiophilicity of the oxygen‐containing functional groups on the surface of diamane. To verify this, 3DP rGO/diamane‐30 was annealed in an Ar/H_2_ atmosphere to remove the functional groups, as evidenced by the FTIR spectrum (Figure S24a, Supporting Information).^[^ ^42^ ^]^ The nucleation overpotentials at various current densities are significantly increased (Figure S24b,c, Supporting Information). Based on the above analysis of the sodiophilicity, electrical conductivity, and specific surface area of electrodes with different diamane contents, the 3DP rGO/diamane‐30 host exhibits the highest CE and lowest nucleation overpotential among the 3DP rGO/diamane hosts. Therefore, 3DP rGO/diamane‐30 host is employed as the representative sample of 3DP rGO/diamane for further characterization and evaluation.

**Figure 3:**
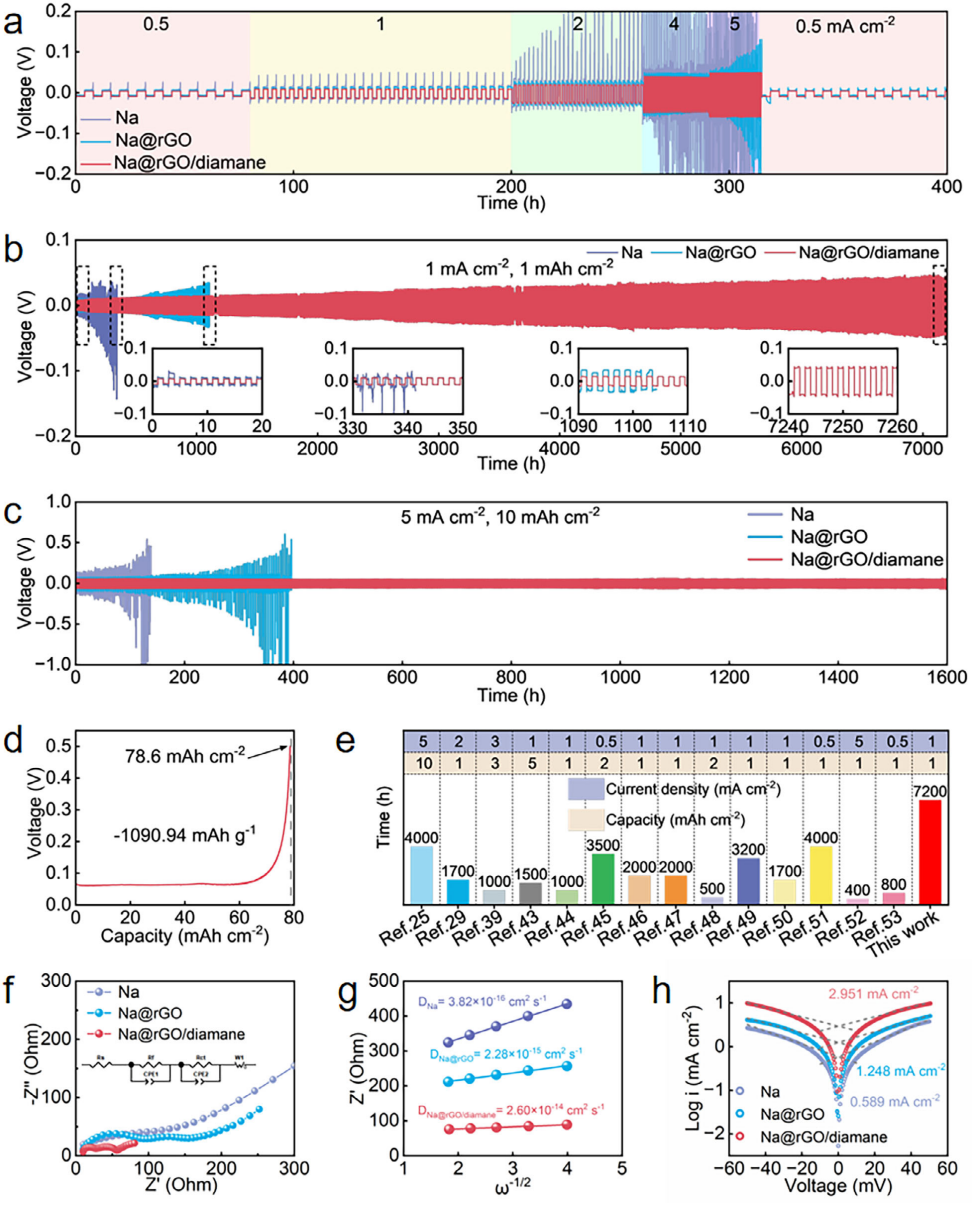
Electrochemical performance evaluation of Na, 3DP Na@rGO, and Na@rGO/diamane anodes. a) Rate capabilities of the symmetric cells based on Na, 3DP Na@rGO, and Na@rGO/diamane anodes. Long‐term Na deposition performance of the Na, 3DP Na@rGO, and Na@rGO/diamane anodes at b) 1 mA cm^−2^ with 1 mAh cm^−2^ and c) 5 mA cm^−2^ with 10 mAh cm^−2^. Inserts in b) are the related magnified voltage profiles at 10, 340, 1100, and 7250 h. d) Stripping curve to evaluate the specific capacity of 3DP Na@rGO/diamane anode at 2 mA cm^−2^. e) Comparison of representative electrochemical performance of carbon‐based nanomaterials for Na metal anodes. f) EIS curves and g) corresponding diffusion coefficients of bare Na, 3DP Na@rGO, and Na@rGO/diamane electrodes after 10 cycles. h) Tafel curves of bare Na, 3DP Na@rGO, and Na@rGO/diamane electrodes.

The electrochemical performance of the 3DP Na@rGO/diamane electrode was evaluated based on the symmetric cell, which is composed of two Na@rGO/diamane electrodes face to face sandwiched with a piece of separator (Celgard 2500). The control samples are Na foil and Na@rGO electrodes. The rate performance of the three electrodes was evaluated at various current densities from 0.5 to 5 mA cm^−2^. As shown in **Figure** **3**a, the 3DP Na@rGO/diamane electrode exhibits the most stable deposition/stripping curves without prominent fluctuation and the lowest voltage hysteresis, achieving 13.3, 22.5, 43.6, 84.9, and 105.4 mV at 0.5, 1, 2, 4, and 5 mA cm^−2^ (Figure S25, Supporting Information). When the current density returns to 0.5 mA cm^−2^, Na@rGO/diamane anode still maintains a low voltage hysteresis of 14.5 mV, proving the reversible and stable deposition/stripping behavior. It is noteworthy that the 3DP Na@rGO electrode can maintain a flat curve and low voltage hysteresis at low current densities, such as 15.4, 29.7, and 58.8 mV at 0.5, 1, and 2 mA cm^−2^, respectively. However, when the current density is increased to 4 or 5 mA cm^−2^, the voltage hysteresis of the Na@rGO electrode is increased to 107.5 or 141.5 mV with an unstable deposition/stripping curve. The bare Na electrode shows an unstable and extremely large voltage hysteresis at high current densities, owing to the formation of Na dendrites and the unstable SEI layer.^[^ ^20^ ^]^ The Na@rGO/diamane electrode shows not only the best rate capability but also the longest lifespan with stable deposition/stripping curve. As shown in Figure 3b, the symmetric cell with 3DP Na@rGO/diamane electrodes is capable of stable cycling for more than 7200 h at 1 mA cm^−2^ with 1 mAh cm^−2^, with the lowest voltage hysteresis of ≈16.0 mV at the initial cycle and slightly increased to ≈85.0 mV at 7200 h. In contrast, the symmetric cells of bare Na and 3DP Na@rGO exhibit reduced stability and higher voltage hysteresis. For example, the voltage hysteresis of the cell with Na electrodes is increased from 25.0 mV at the initial cycle sharply to ≈150.0 mV after 300 h. The deposition/stripping curve of the Na electrode is unstable, and the cell is short‐circuited after ≈300 h of operation. The possible reason can be attributed to the formation of Na dendrites and an unstable SEI layer. Although the voltage hysteresis of the cell with the Na@rGO electrodes is merely 20.0 mV at the initial cycle, it is gradually increased to 69.0 mV after 1100 h of operation. The 3DP Na@rGO/diamane electrode also exhibits long‐cycle stability at high current density with large areal capacity. As shown in Figure 3c, when the current density and capacity are increased to 5 mA cm^−2^ and 10 mAh cm^−2^, the symmetric cell with 3DP Na@rGO/diamane electrodes can still cycle for more than 1600 h with a minimal voltage hysteresis of ≈110 mV. Conversely, the symmetric cells of bare Na and the 3DP Na@rGO electrodes can only cycle for ≈140 and 400 h at the same condition, and then the voltage hysteresis increases sharply and subsequently short circuit. Moreover, the 3DP Na@rGO/diamane anode also delivers an ultra‐high capacity of 78.60 mAh cm^−2^ (≈1090.94 mAh g^−1^, 93.64% of the theoretical specific capacity of the Na metal anode) at 2 mA cm^−2^, proving that the designed thick electrode with enough space can store a large amount of sodium (Figure 3d). To underscore the superiority of the 3DP rGO/diamane electrode, a comparison was made with various representative carbonaceous hosts for sodium metal anodes (Figure 3e, Table S1, Supporting Information).^[^ ^25^, ^29^, ^39^, ^43^, ^44^, ^45^, ^46^, ^47^, ^48^, ^49^, ^50^, ^51^, ^52^, ^53^ ^]^ Our designed 3DP rGO/diamane electrode exhibits outstanding performance with the exceptional long cycle stability of 7200 h at 1 mA cm^−2^ with 1 mAh cm^−2^.

### 2.3 Mechanism Investigation

To explore the mechanism of the high performance of 3DP Na@rGO/diamane electrode, the surface kinetics including the charge transfer resistance (*R*_ct_), the diffusion coefficient (*D*) of Na ions, and the exchange current density were explored by measuring the electrochemical impedance spectra (EIS) before and after 10 cycles and Tafel plots of all electrodes, respectively (Figure 3f–h, Figure S26, Supporting Information). The EIS equivalent circuit is shown in the insert of Figure 3f. As shown from Table S2 (Supporting Information), the *R*_ct_ of all electrodes after 10 cycles is decreased compared with that before cycling due to the formation of the SEI which can speed up charge transfer and decrease the *R*_ct_.^[^ ^54^, ^55^ ^]^ The *R*_ct_ of the 3DP rGO/diamane electrode is decreased from 56.68 Ω before cycles to 29.64 Ω after 10 cycles, which is the smallest one among various electrodes. The smallest *R*_ct_ suggests the fastest charge transfer speed and the formation of a stable SEI film on the designed 3DP Na@rGO/diamane electrode.^[^ ^56^ ^]^ The diffusion coefficient of Na ions in the 3DP rGO/diamane electrode is 2.60 × 10^−14^ cm^2^ s^−1^, which is the highest among various electrodes shown in Figure 3g and Table S2 (Supporting Information). From the Tafel curves shown in Figure 3h, the exchange current density of the 3DP Na@rGO/diamane electrode is 2.951 mA cm^−2^, which is much higher than that of Na (0.589 mA cm^−2^) and Na@rGO electrode (1.248 mA cm^−2^), indicating that the electrochemical kinetics of 3DP rGO/diamane electrode is enhanced by the incorporation of the diamane.^[^ ^57^ ^]^

The mechanism of the excellent performance of the 3DP rGO/diamane electrode was further investigated by examining the deposition morphology, current density distribution, and the chemical composition of SEI layer. The morphological evolution of various electrodes during Na deposition/stripping was systematically investigated by the ex‐situ SEM technique as shown in Figures S27–S29 (Supporting Information). For the Na foil electrode, Na dendrites formed promptly on the surface unevenly and eventually aggregated into large dendrite blocks with increasing of deposition amounts from 1 to 5 mAh cm^−2^ at 1 mA cm^−2^ (Figure S27b–d, Supporting Information). Then after stripping with a capacity of 5 mAh cm^−2^ at 1 mA cm^−2^, there are plenty of irregular cavities on the surface of Na foil, which leads to uneven charge distribution and abnormal sodium ions flux (Figure S27e–g, Supporting Information).^[^ ^58^ ^]^ In addition, the Na@rGO electrode exhibits a few unevenly distributed dendrites during deposition/stripping processes (Figure S28, Supporting Information). In contrast, the surface of Na@rGO/diamane electrode always keeps smooth and dendrite‐free morphology during the whole deposition/stripping processes (Figure S29, Supporting Information), which indicates that Na ions can be uniformly deposited and stripped on the surface of the electrode with the guidance of diamane.^[^ ^59^, ^60^ ^]^

**Figure 4:**
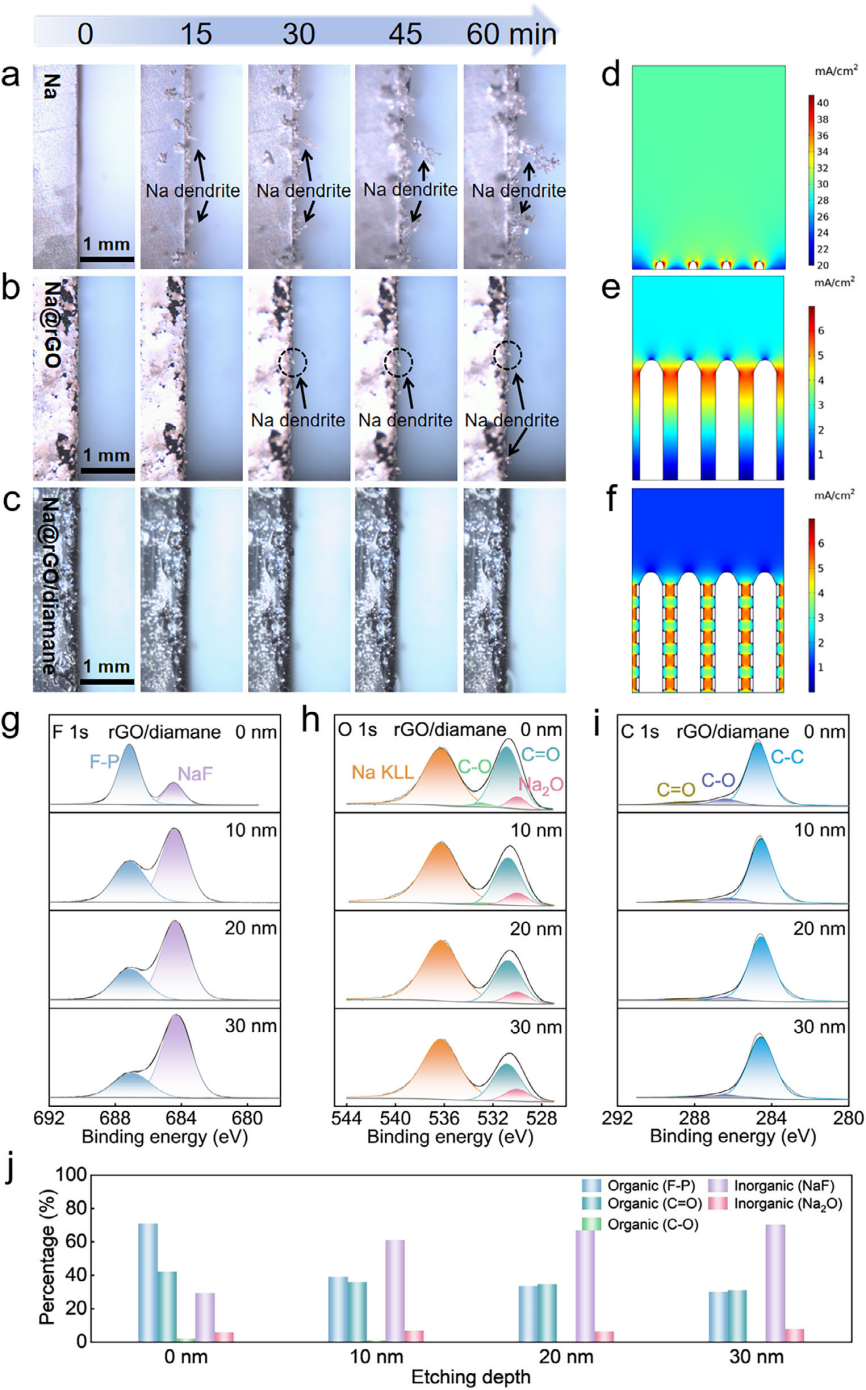
In‐situ optical microscopy images of Na deposited on a) Na foil, b) 3DP Na@rGO, and c) Na@rGO/diamane electrodes at 5 mA cm^−2^ for 60 min. Surface current density distribution on d) Na, e) 3DP Na@rGO and f) 3DP Na@rGO/diamane anodes. XPS depth profiles of g) F 1s, h) O 1s, and i) C 1s of 3DP rGO/diamane electrode after 10 cycles at 2 mA cm^−2^ with 1 mAh cm^−2^. j) The content comparison of the organic and inorganic species at different depths.

The Na deposition behavior on various electrodes at the millimeter scale was further explored by in‐situ optical microscopy characterization. At 5 mA cm^−2^, Na ions clearly nucleated on the surface of the Na electrode just after ≈2 min (Figure S30, Supporting Information). Dendrites grow on the surface of the Na electrode at 15 min of deposition. After 60 min of deposition, the unrestricted growth of dendrites contact with the counter electrode and lead to the short‐circuit (**Figure** **4**a). For the 3DP Na@rGO electrode, a few mossy‐like dendrites appear at 45 min of deposition (Figure 4b). In contrast, there was no dendrite on the surface of the 3DP Na@rGO/diamane electrode for 1 h of deposition (Figure 4c). Therefore, the 3DP Na@rGO/diamane electrode can effectively inhibit the formation of dendrites and promote uniform deposition. The main reason for this result is that the diamane can guide the uniform nucleation of Na ions and promote lateral deposition, thus avoiding the formation of Na dendrites.^[^ ^61^, ^62^ ^]^

The favorable deposition morphology of the 3DP Na@rGO/diamane is related to the local current density. Therefore, the local current densities of the electrodes were simulated using COMSOL Multiphysics, as shown in Figure 4d–f. The “hotspots” are clearly observed on the surface of Cu (Figure 4d), which leads to large local current density and uneven distribution. On the contrary, the local current density of 3DP rGO is greatly reduced due to its large specific surface area (Figure 4e). According to Sand's time formula, dendrite nucleation time is inversely proportional to the square of the local current density.^[^ ^20^ ^]^ Therefore, the large specific surface area can lead to dendrite‐free morphology easily. However, there are still some “hotspots” on the rGO sheets due to the “tip effect”, with the sodiophilicity of the rGO sheets require high nucleation overpotential, the dendrites are easily formed at large current density condition.^[^ ^63^ ^]^ In contrast, the local current density on the surface of 3DP rGO/diamane electrode is more even and lower (Figure 4f), leading to the dendrite‐free morphology during repeated cycles.

The excellent performance is also related to the SEI layer. The SEI compositions of different electrodes were analyzed by in‐depth XPS technique at a total depth of 30 nm as shown in Figure 4g–j, Figures S31 and S32 (Supporting Information). As shown in Figure 4g–i, the signals of F 1s, O 1s, and C 1s are gradually changed with the depth of SEI layer. The organic species of the SEI layer of 3DP rGO/diamane electrode are predominately distributed at the outer layer of SEI, which is reduced with the depth of SEI layer. While the inorganic species, such as NaF, is increased with the depth of SEI layer, especially in the inner layer of SEI as shown in Figure 4j. It is reported that the inorganic NaF is critical for the stable SEI layer to protect the Na metal.^[^ ^64^ ^]^ The inorganic species of the SEI layer of 3DP rGO/diamane electrode at the inner layer (30 nm) are more than that of 3DP rGO and Cu electrodes (Figure 4j, Figures S31 and S32, Supporting Information). This is one of the reasons for the electrochemical performance of 3DP rGO/diamane electrode is better than 3DP rGO and Cu electrodes.^[^ ^22^, ^65^ ^]^ In other words, the NaF of the SEI layer, as a pivotal inorganic species, is vital for the stable SEI layer to protect the anode and enable the long‐cycle lifespan.^[^ ^66^, ^67^ ^]^

### 2.4 Electrochemical Performance Evaluation at High Temperature

**Figure 5:**
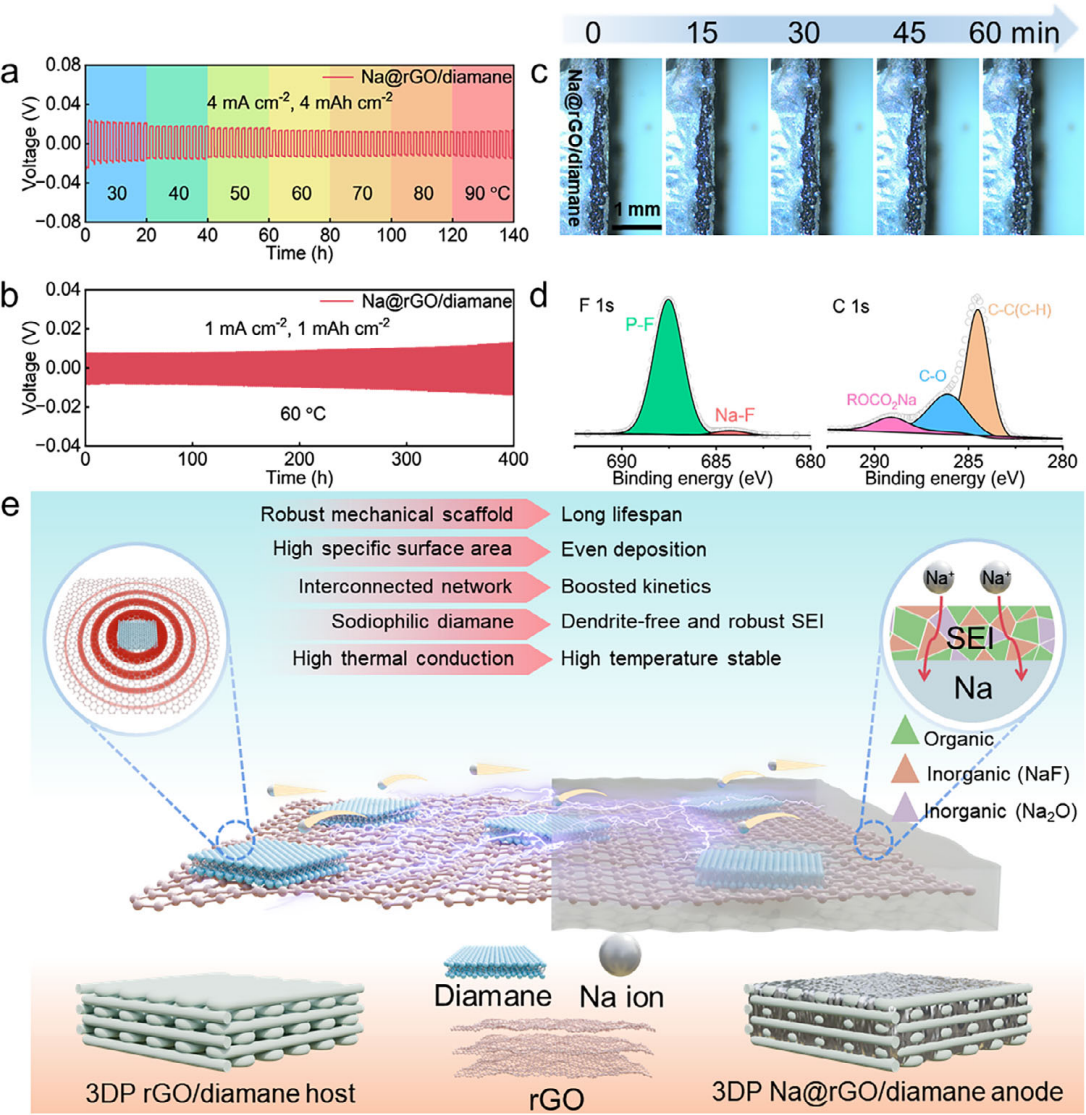
a) Deposition/stripping curves at different temperatures from 30 to 90 °C at 4 mA cm^−2^ with 4 mAh cm^−2^. b) Long‐term cycling performance at 1 mA cm^−2^ with 1 mAh cm^−2^ at 60 °C. c) 3DP Na@rGO/diamane deposition morphology evolution measured by in‐situ optical microscopy at 5 mA cm^−2^ for 1 h at 60 °C. d) High‐resolution XPS: F 1s and C 1s profiles of the 3DP rGO/diamane electrode after 10 cycles at 60 °C. e) Schematic illustration of the 3DP Na@rGO/diamane anode with porous framework to enhance the performance of Na metal anode with a robust SEI layer.

Moreover, our 3DP Na@rGO/diamane anode also exhibits excellent performance at elevated temperatures. The symmetric cells with Na, 3DP Na@rGO, and 3DP Na@rGO/diamane electrodes were measured at 4 mA cm^−2^ with 4 mAh cm^−2^ at temperatures ranging from 30 to 90 °C (**Figures** **5**a, S33, Supporting Information). The result reveals that the Na@rGO/diamane electrode shows stable cycling at different temperatures, and the voltage hysteresis is decreased with increasing temperature (Figure S34, Supporting Information). For example, the voltage hysteresis of the Na@rGO/diamane symmetric cell decreases from 41.0 mV at 30 °C to 25.8 mV at 90 °C. The reason is that the nucleation energy is reduced at higher temperatures due to the thermal activation.^[^ ^68^ ^]^ On the contrary, the Na and Na@rGO cells short‐circuit when the temperature rises to 40 and 50 °C, respectively (Figure S33, Supporting Information). Different from the Na@rGO/diamane, the voltage hysteresis of Na and Na@rGO shows an unstable and even increasing trend with the increase of temperature (Figure S34, Supporting Information), probably because the serious side reactions are generated and the unstable SEI layers were formed on the surface of Na and 3DP Na@rGO electrodes at elevated temperatures.^[^ ^69^ ^]^ Benefiting from the excellent thermal property of diamane, the symmetric cell with 3DP Na@rGO/diamane electrodes can stably cycle for 400 h with a low voltage hysteresis of 15.0 mV at 1 mA cm^−2^ with 1 mAh cm^−2^ at 60 °C (Figure 5b). The morphology evolution of different electrodes at 5 mA cm^−2^ with 5 mAh cm^−2^ at 60 °C is explored by the in‐situ optical microscopy (Figures 5c, S35, Supporting Information). Na dendrites grow rapidly on the Na foil and cause short‐circuits in less than 1 h (Figure S35a, Supporting Information). Similarly, the Na dendrites grow slowly and steadily on the 3DP Na@rGO electrode for 1 h (Figure S35b, Supporting Information). Even worse, the side reaction on the surface of the electrodes of Na and Na@rGO becomes more severe at high temperature, resulting in a large number of bubbles. In contrast, there are no dendrites or bubbles generated on the surface of the 3DP Na@rGO/diamane during 1 h of deposition (Figure 5c). The above results confirm that the application of diamane can significantly improve the electrochemical performance of the electrode even at a high temperature.

The surface chemical composition and properties of Cu, 3DP rGO, and 3DP rGO/diamane electrodes after 10 cycles at 60 °C were also investigated by ex‐situ XPS analysis. As shown in Figure 5d, Figures S36 and S37 (Supporting Information), affected by severe side reaction because of high temperature and unstable SEI layer, the NaF contents of Cu and 3DP rGO electrodes are much less than that of 3DP rGO/diamane electrode, which is crucial for the cycle stability and cycle life.^[^ ^70^ ^]^ In contrast, even under high temperatures, the 3DP rGO/diamane electrode can form a stable and inorganic species (NaF) rich SEI layer due to the excellent thermal and sodiophilic properties of diamane.

The exceptional electrochemical characteristics of the Na@rGO/diamane are primarily ascribed to a multitude of beneficial attributes (Figure 5e). First, the artificial porous structure acts as a robust mechanical scaffold that sustains the electrode during repeated cycling. Second, the rGO/diamane host with elevated specific surface area reduces the local current density, thereby extending the induction period for dendrite formation and providing optimal sites for sodium ion nucleation and subsequent deposition. Third, the interconnected network of rGO avoids the binders and additives and ensures superior electrical conductivity, and promotes efficient charge transfer dynamics. Fourth, the selected diamane nanoflakes exhibit pronounced sodiophilicity, which is instrumental in achieving dendrite‐free deposition morphology. Additionally, the stable SEI layer rich with NaF is formed on the surface of the Na@rGO/diamane anode. This SEI layer facilitates the uniform deposition of sodium, effectively mitigating the proliferation of dendrite growth and fostering dendrite‐free deposition morphology. Lastly, the diamane on the rGO sheets has a high thermal conductivity which can effectively disperse the heat generated during cycles, so as to adapt to a high‐temperature environment. As a result, the anode with the integrated SEI layer demonstrates an extraordinarily long cycle life and high CE, even at elevated temperature condition of 60 °C.

### 2.5 Full Cell Application

**Figure 6:**
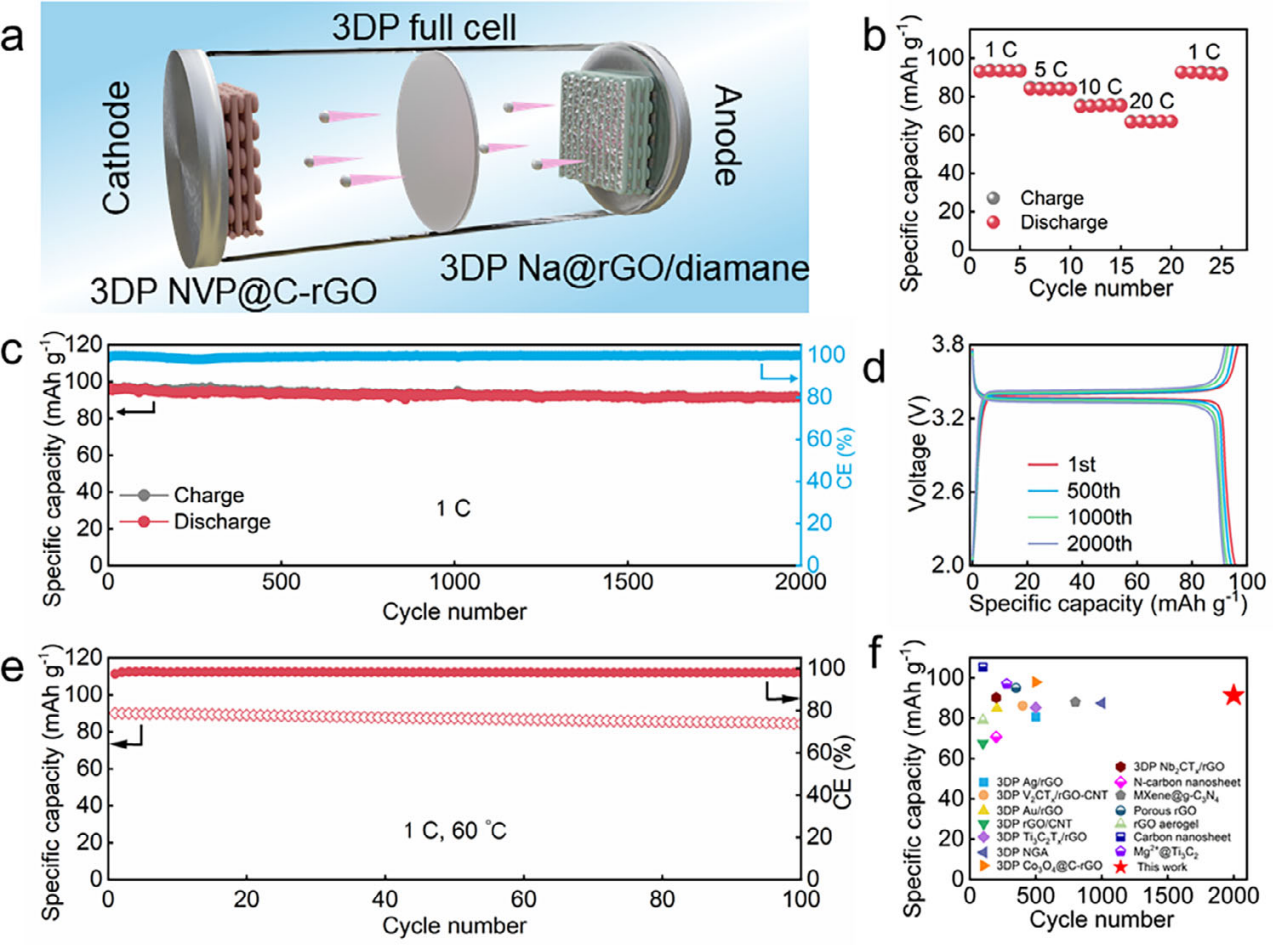
Electrochemical performance evaluation of 3DP NVP@C‐rGO||3DP Na@rGO/diamane full cell. a) Schematic diagram. b) Rate capability. c) Cycling performance and corresponding CE at 1 C. d) Galvanostatic charge–discharge (GCD) curves at the 1st, 500th, 1000th, and 2000th cycles at 1 C. e) Cycling performance at 1 C with the corresponding CE at 60 °C. f) Performance comparison with various 2D nanomaterial‐based Na metal batteries.

To evaluate the practical application of the 3DP Na@rGO/diamane anode, a full battery composed of 3DP NVP@C‐rGO cathode and 3DP Na@rGO/diamane anode was assembled (**Figure** **6**a). The full cell delivers the capacities of 93.15, 83.87, 75.17, and 66.87 mAh g^−1^ at 1, 5, 10, and even 20 C (1 C = 117 mA g^−1^) (Figure 6b, Figure S38, Supporting Information), which are much higher than those of 3DP NVP@C‐rGO||Na and 3DP NVP@C‐rGO||3DP Na@rGO full cells at various current densities (Figure S39 and Table S3, Supporting Information). Moreover, the full cell with Na@rGO/diamane anode can deliver an ultra‐high specific capacity of 91.37 mAh g^−1^ after 2000 cycles with a minimal capacity decay rate of 0.0027% per cycle at 1 C. Concurrently, the full cell has maintained an average high CE of 99.58% (Figure 6c). The voltage curves from the 1st cycle to 2000th cycle are overlapped and the voltage hysteresis remains almost constant (Figure 6d). In contrast, the full cells with Na and 3DP Na@rGO anodes exhibit a shorter life, faster capacity decay (Figure S40, Supporting Information), and larger voltage hysteresis (Figure S41, Supporting Information). Furthermore, an optimized N/P ratio of 1.8 was prepared by electrodepositing 0.11 mAh cm^−2^ Na at 0.1 mA cm^−2^ into the Cu, 3DP rGO, and rGO/diamane to form the Na@Cu, Na@rGO and Na@rGO/diamane anodes. The full cells with Na@Cu and Na@rGO anodes can only deliver capacities of 1.97 and 4.37 mAh g^−1^ at the 29th and 78th cycles at 1 C, respectively (Figure S42a,b, Supporting Information). In contrast, the full cell with Na@rGO/diamane anode can stably cycle for more than 200 cycles with a high reversible specific capacity of 94.15 mAh g^−1^ at 1 C, as shown in Figure S42c (Supporting Information). In addition, this full cell also delivers a high energy density of 86.13 Wh kg^−1^. Moreover, the full cell with Na@rGO/diamane anode can also deliver a high capacity of 84.38 mAh g^−1^ after 100 cycles at 1 C, with an average CE of 98.12% evaluated at a high temperature of 60 °C (Figure 6e). Additionally, we have compared our results with other full cells with 2D material‐reinforced metal anodes (Figure 6f, Table S4, Supporting Information).^[^ ^19^, ^20^, ^21^, ^22^, ^23^, ^29^, ^30^, ^46^, ^47^, ^52^, ^71^, ^72^, ^73^, ^74^ ^]^ The result shows that our dedicated full cell with 3DP Na@rGO/diamane anode exhibits a much improved lifespan with a high capacity of 91.37 mAh g^−1^ after cycles.

## 3 Conclusion

In summary, a hierarchical 3DP rGO/diamane microlattice aerogel was successfully fabricated by the DIW 3D printing technology and utilized as a host for Na metal anode. The 3DP rGO/diamane microlattice aerogel possesses a hierarchical porous structure at both macro and micro levels, which significantly amplifies the specific surface area and reduces the local current density. In addition, the diamane nanoflakes distributed uniformly on the rGO sheets act as the active sites to guide the Na metal nucleation and uniform deposition, enabling dendrite‐free morphology. With the advantages of porosity and sodiophilicity, the 3DP Na@rGO/diamane anode can deliver an ultra‐high areal capacity of 78.60 mAh cm^−2^ (1090.94 mAh g^−1^), and can maintain stable cycling of more than 7200 h at 1 mA cm^−2^ with 1 mAh cm^−2^. It also operates continuously for >1600 h at 5 mA cm^−2^ with 10 mAh cm^−2^, demonstrating robust stability at high current density and large areal capacity. Furthermore, the 3DP Na@rGO/diamane enhances the long‐term stability even at an elevated operation temperature of 60 °C, sustaining a prolonged cycle lifespan of 400 h at 1 mA cm^−2^ with 1 mAh cm^−2^. Finally, the 3DP NVP@C‐rGO||3DP Na@rGO/diamane full cell demonstrated an ultra‐long cycle life of more than 2000 cycles with a low capacity decay rate of 0.0027% per cycle at 1 C.

## Conflict of Interest

The authors declare no conflict of interest.

## Supporting information



Supporting Information

Supplemental Video 1

Supplemental Video 2

## Data Availability

The data that support the findings of this study are available from the corresponding author upon reasonable request.

## References

[1] C. Zhao , Q. Wang , Z. Yao , J. Wang , B. Sánchez‐Lengeling , F. Ding , X. Qi , Y. Lu , X. Bai , B. Li , H. Li , A. Aspuru‐Guzik , X. Huang , C. Delmas , M. Wagemaker , L. Chen , Y.‐S. Hu , Science 2020, 370, 708. 33154140 10.1126/science.aay9972

[2] Y. Li , Q. Zhou , S. Weng , F. Ding , X. Qi , J. Lu , Y. Li , X. Zhang , X. Rong , Y. Lu , X. Wang , R. Xiao , H. Li , X. Huang , L. Chen , Y.‐S. Hu , Nat. Energy 2022, 7, 511.

[3] X. Zheng , L. Huang , X. Ye , J. Zhang , F. Min , W. Luo , Y. Huang , Chem 2021, 7, 2312.

[4] M. Han , C. Zhu , T. Ma , Z. Pan , Z. Tao , J. Chen , Chem. Commun. 2018, 54, 2381. 10.1039/c7cc09751d 29450421

[5] Y. Zhao , K. R. Adair , X. Sun , Energy Environ. Sci. 2018, 11, 2673.

[6] H. Wang , D. Yu , C. Kuang , L. Cheng , W. Li , X. Feng , Z. Zhang , X. Zhang , Y. Zhang , Chem 2019, 5, 313.

[7] D. Lin , Y. Liu , Y. Cui , Nat. Nanotechnol. 2017, 12, 194. 28265117 10.1038/nnano.2017.16

[8] B. Lee , E. Paek , D. Mitlin , S. W. Lee , Chem. Rev. 2019, 119, 5416. 30946573 10.1021/acs.chemrev.8b00642

[9] C. Bao , B. Wang , P. Liu , H. Wu , Y. Zhou , D. Wang , H. Liu , S. Dou , Adv. Funct. Mater. 2020, 30, 2004891.

[10] X. Xia , S. Xu , F. Tang , Y. Yao , L. Wang , L. Liu , S. He , Y. Yang , W. Sun , C. Xu , Y. Feng , H. Pan , X. Rui , Y. Yu , Adv. Mater. 2023, 35, 2209511. 10.1002/adma.202209511 36576022

[11] C. Wang , Y. Zheng , Z. N. Chen , R. Zhang , W. He , K. Li , S. Yan , J. Cui , X. Fang , J. Yan , G. Xu , D. Peng , B. Ren , N. Zheng , Adv. Energy Mater. 2023, 13, 2204125.

[12] M. Moorthy , B. Moorthy , B. K. Ganesan , A. Saha , S. Yu , D. H. Kim , S. Hong , S. Park , K. Kang , R. Thangavel , Y. S. Lee , Adv. Funct. Mater. 2023, 33, 2300135.

[13] J. Zhang , Y. Wang , Q. Xia , X. Li , B. Liu , T. Hu , M. Tebyetekerwa , S. Hu , R. Knibbe , S. Chou , Angew. Chem., Int. Ed. 2024, 63, 202318822. 10.1002/anie.202318822 38372507

[14] Y. Hu , J. Fu , X. Lin , J. Xu , J. Luo , F. Zhao , Y. Liu , W. Li , J. T. Kim , H. Su , X. Hao , H. Ren , M. Yang , Y. Huang , X. Sun , Matter 2024, 7, 1018.

[15] G. Sun , C. Lou , B. Yi , W. Jia , Z. Wei , S. Yao , Z. Lu , G. Chen , Z. Shen , M. Tang , F. Du , Nat. Commun. 2023, 14, 6501. 37845205 10.1038/s41467-023-42308-0 PMC10579357

[16] P. Liu , H. Yi , S. Zheng , Z. Li , K. Zhu , Z. Sun , T. Jin , L. Jiao , Adv. Energy Mater. 2021, 11, 2101976.

[17] X. Lai , Z. Xu , X. Yang , Q. Ke , Q. Xu , Z. Wang , Y. Lu , Y. Qiu , Adv. Energy Mater. 2022, 12, 2103540.

[18] H. Huang , Y. Wang , M. Li , H. Yang , Z. Chen , Y. Jiang , S. Ye , Y. Yang , S. He , H. Pan , X. Wu , Y. Yao , M. Gu , Y. Yu , Adv. Mater. 2023, 35, 2210826. 10.1002/adma.202210826 36731534

[19] D. Pan , H. Yang , Y. Liu , H. Wang , T. Xu , D. Kong , J. Yao , Y. Shi , X. Li , H. Y. Yang , Y. Wang , Nanoscale 2023, 15, 17482. 37861463 10.1039/d3nr03046f

[20] Y. Liu , H. Wang , H. Yang , Z. Wang , Z. Huang , D. Pan , Z. Zhang , Z. Duan , T. Xu , D. Kong , X. Li , Y. Wang , J. Sun , ACS Nano 2023, 17, 10844. 37204014 10.1021/acsnano.3c02506

[21] W. Bai , H. Wang , D. H. Min , J. Miao , B. Li , T. Xu , D. Kong , X. Li , X. Yu , Y. Wang , H. S. Park , Adv. Sci. 2024, 11, 2404419. 10.1002/advs.202404419 PMC11425270 39018250

[22] Y. Liu , H. Wang , D. Pan , J. Hou , J. Yao , D. Kong , T. Xu , Y. Shi , X. Li , H. Y. Yang , Y. Wang , Z. S. Wu , Adv. Funct. Mater. 2024, 34, 2405460.

[23] H. Wang , W. Bai , H. Wang , D. Kong , T. Xu , Z. Zhang , J. Zang , X. Wang , S. Zhang , Y. Tian , X. Li , C.‐S. Lee , Y. Wang , Energy Storage Mater. 2023, 55, 631.

[24] F. Lavini , M. Rejhon , E. Riedo , Nat. Rev. Mater. 2022, 7, 814.

[25] T. Li , J. Sun , S. Gao , B. Xiao , J. Cheng , Y. Zhou , X. Sun , F. Jiang , Z. Yan , S. Xiong , Adv. Energy Mater. 2021, 11, 2003699.

[26] Y. Liu , Y.‐K. Tzeng , D. Lin , A. Pei , H. Lu , N. A. Melosh , Z.‐X. Shen , S. Chu , Y. Cui , Joule 2018, 2, 1595.

[27] P. B. Sorokin , B. I. Yakobson , Nano Lett. 2021, 21, 5475. 34213910 10.1021/acs.nanolett.1c01557

[28] S. Dai , Z. Lin , H. Hu , Y. Wang , L. Zeng , Appl. Phys. Rev. 2024, 11, 041319.

[29] J. Yan , G. Zhi , D. Kong , H. Wang , T. Xu , J. Zang , W. Shen , J. Xu , Y. Shi , S. Dai , X. Li , Y. Wang , J. Mater. Chem. A 2020, 8, 19843.

[30] Z. Wang , Z. Huang , H. Wang , W. Li , B. Wang , J. Xu , T. Xu , J. Zang , D. Kong , X. Li , H. Y. Yang , Y. Wang , ACS Nano 2022, 16, 9105. 35666854 10.1021/acsnano.2c01186

[31] H. W. Aijiao Li , X. Liu , W. Shen , C. Fang , Z. Zhang , Y. Zhang , L. Chen , Q. Wang , B. Wan , Y.e Wang , C. Shan , Chem. Eng. J. 2024, 491, 151914.

[32] L.‐S. Fan , L. Constantin , D.‐w. Li , L. Liu , K. Keramatnejad , C. Azina , X. Huang , H. R. Golgir , Y. Lu , Z. Ahmadi , F. Wang , J. Shield , B. Cui , J.‐F. Silvain , Y.‐F. Lu , Light Sci. Appl. 2017, 7, 17177. 10.1038/lsa.2017.177 PMC6060054 30839522

[33] T. Petit , L. Puskar , Diamond Relat. Mater. 2018, 89, 52.

[34] Y. Wang , Y. V. Lim , S. Huang , M. Ding , D. Kong , Y. Pei , T. Xu , Y. Shi , X. Li , H. Y. Yang , Nanoscale 2020, 12, 4341. 31994571 10.1039/c9nr09278a

[35] B. Tian , Z. Huang , H. Yang , H. Wang , T. Xu , D. Kong , C. Gao , J. Zang , X. Li , Y. Wang , Ionics 2022, 28, 4641.

[36] K. Chen , X. Li , J. Zang , Z. Zhang , Y. Wang , Q. Lou , Y. Bai , J. Fu , C. Zhuang , Y. Zhang , L. Zhang , S. Dai , C. Shan , Nanoscale 2021, 13, 12370. 34254619 10.1039/d1nr02158c

[37] J. Miao , Y. Fang , H. Wang , L. Lyu , W. Bai , B. Li , D. Kong , T. Xu , X. Li , Z.‐L. Xu , Y. Wang , Energy Storage Mater. 2024, 71, 103591.

[38] X. Hu , Z. Li , Y. Zhao , J. Sun , Q. Zhao , J. Wang , Z. Tao , J. Chen , Sci. Adv. 2017, 3, 1602396. 10.1126/sciadv.1602396 PMC5287700 28164158

[39] N. Mubarak , F. Rehman , M. Ihsan‐Ul‐Haq , M. Xu , Y. Li , Y. Zhao , Z. Luo , B. Huang , J. K. Kim , Adv. Energy Mater. 2022, 12, 2103904.

[40] X. Li , J. Fu , Y. Sun , M. Sun , S. Cheng , K. Chen , X. Yang , Q. Lou , T. Xu , Y. Shang , J. Xu , Q. Chen , C. Shan , Nanoscale 2019, 11, 13343. 31271407 10.1039/c9nr03581h

[41] J. Yun , B.‐K. Park , E.‐S. Won , S. H. Choi , H. C. Kang , J. H. Kim , M.‐S. Park , J.‐W. Lee , ACS Energy Lett. 2020, 5, 3108.

[42] J. C. Yoon , X. Dai , K. N. Kang , J. Hwang , M. J. Kwak , F. Ding , J. H. Jang , ACS Nano 2021, 15, 11655. 34196523 10.1021/acsnano.1c02178

[43] Z. Sun , Y. Ye , J. Zhu , E. Zhou , J. Xu , M. Liu , X. Kong , S. Jin , H. Ji , Small 2022, 18, 2107199. 10.1002/smll.202107199 35373497

[44] Y. Yu , Z. Wang , Z. Hou , W. Ta , W. Wang , X. Zhao , Q. Li , Y. Zhao , Q. Zhang , Z. Quan , ACS Appl. Energy Mater. 2019, 2, 3869.

[45] H. Ye , C.‐Y. Wang , T.‐T. Zuo , P.‐F. Wang , Y.‐X. Yin , Z.‐J. Zheng , P. Wang , J. Cheng , F.‐F. Cao , Y.‐G. Guo , Nano Energy 2018, 48, 369.

[46] K. Yan , S. Zhao , J. Zhang , J. Safaei , X. Yu , T. Wang , S. Wang , B. Sun , G. Wang , Nano Lett. 2020, 20, 6112. 32633528 10.1021/acs.nanolett.0c02215

[47] Y. Xie , Z. Han , H. Li , J. Hu , L. Zhang , A. Wang , S. Chang , J. Xu , C. Liu , Y. Lai , Z. Zhang , Chem. Eng. J. 2022, 427, 130959.

[48] S. S. Chi , X. G. Qi , Y. S. Hu , L. Z. Fan , Adv. Energy Mater. 2018, 8, 1702764.

[49] X. Ji , Z. Lin , J. Zeng , Y. Lin , Y. Mu , S. Wang , Z. Ren , J. Yu , Carbon 2020, 158, 394.

[50] J. Liang , W. Wu , L. Xu , X. Wu , Carbon 2021, 176, 219.

[51] C. Chu , N. Wang , L. Li , L. Lin , F. Tian , Y. Li , J. Yang , S.‐x. Dou , Y. Qian , Energy Storage Mater. 2019, 23, 137.

[52] F. Wu , J. Zhou , R. Luo , Y. Huang , Y. Mei , M. Xie , R. Chen , Energy Storage Mater. 2019, 22, 376.

[53] Y.‐J. Kim , J. Lee , S. Yuk , H. Noh , H. Chu , H. Kwack , S. Kim , M.‐H. Ryou , H.‐T. Kim , J. Power Sources 2019, 438, 227005.

[54] X. Li , C. Zhuang , J. Xu , L. Li , T. Xu , S. Dai , X. Wang , X. Li , Y. Wang , Nanoscale 2021, 13, 8199. 33885119 10.1039/d1nr00993a

[55] Z. Huang , Z. Wang , X. Wang , S. Zhang , T. Xu , Z. Zhang , J. Zang , D. Kong , X. Li , Y. Wang , Solid State Ionics 2022, 380, 115941.

[56] L. Hou , L. Zhang , J. Zang , W. Shen , T. Zhang , X. Huang , H. Yuan , D. Kong , Y. Wang , X. Li , T. Xu , J. Phys. D: Appl. Phys. 2022, 55, 234002.

[57] H. Zhao , C. Zhuang , J. Xu , Z. Zhang , W. Shen , H. Tang , Y. Wang , T. Xu , X. Wang , X. Li , Ionics 2020, 26, 5019.

[58] L. Yue , Y. Qi , Y. Niu , S. Bao , M. Xu , Adv. Energy Mater. 2021, 11, 2102497.

[59] W. Cao , M. Liu , W. Song , Z. Li , B. Li , P. Wang , A. Fisher , J. Niu , F. Wang , Adv. Sci. 2024, 11, 202402321. 10.1002/advs.202402321 PMC11336894 38889333

[60] S. Xia , W. Fan , Z. Hou , C. Li , Z. Jiang , J. Yang , J. Mao , S. Zheng , Adv. Funct. Mater. 2024, 34, 2314954.

[61] F. Huang , P. Xu , G. Fang , S. Liang , Adv. Mater. 2024, 36, 2405310. 10.1002/adma.202405310 39152941

[62] X. Li , X. Zhang , J. Xu , Z. Duan , Y. Xu , X. Zhang , L. Zhang , Y. Wang , P. K. Chu , Adv. Sci. (Weinh) 2024, 11, 2305467. 38059813 10.1002/advs.202305467 PMC10837388

[63] C.‐P. Yang , Y.‐X. Yin , S.‐F. Zhang , N.‐W. Li , Y.‐G. Guo , Nat. Commun. 2015, 6, 8058. 26299379 10.1038/ncomms9058 PMC4560781

[64] L. Gao , J. Chen , Q. Chen , X. Kong , Sci. Adv. 2022, 8, abm4606. 10.1126/sciadv.abm4606 PMC8836821 35148184

[65] B. Yang , Z. Yang , X. Zhang , Y. Zhao , Y. Wang , J. Chen , S. Wang , Q. Zhao , Z. Hou , Adv. Funct. Mater. 2024, 34, 2407783.

[66] C. S. Santos , A. Botz , A. S. Bandarenka , E. Ventosa , W. Schuhmann , Angew. Chem., Int. Ed. 2022, 61, 202202744. 10.1002/anie.202202744 PMC9322322 35312219

[67] C. Zhu , D. Wu , Z. Wang , H. Wang , J. Liu , K. Guo , Q. Liu , J. Ma , Adv. Funct. Mater. 2023, 34, 2214195.

[68] Y. Sun , J.‐C. Li , H. Zhou , S. Guo , Energy Environ. Sci. 2023, 16, 4759.

[69] J. Wang , W. Huang , A. Pei , Y. Li , F. Shi , X. Yu , Y. Cui , Nat. Energy 2019, 4, 664.

[70] H. Wang , J. Wang , W. Li , J. Hu , J. Dong , D. Zhai , F. Kang , Adv. Mater. 2024, 36, 2409062. 10.1002/adma.202409062 39240064

[71] H. Yang , H. Wang , W. Li , B. Tian , T. Xu , D. Kong , S. Huang , K. Liu , X. Li , H. Y. Yang , Y. Wang , J. Mater. Chem. A 2022, 10, 16842.

[72] C. Bao , J. Wang , B. Wang , J. Sun , L. He , Z. Pan , Y. Jiang , D. Wang , X. Liu , S. X. Dou , J. Wang , ACS Nano 2022, 16, 17197. 36222585 10.1021/acsnano.2c07771

[73] B. Huang , S. Sun , J. Wan , W. Zhang , S. Liu , J. Zhang , F. Yan , Y. Liu , J. Xu , F. Cheng , Y. Xu , Y. Lin , C. Fang , J. Han , Y. Huang , Adv. Sci. 2023, 10, 2206845. 10.1002/advs.202206845 PMC10104674 36793148

[74] H. Jiang , X. Lin , C. Wei , Y. Zhang , J. Feng , X. Tian , Small 2022, 18, 2107637. 10.1002/smll.202107637 35315554

