# Supplementary material for: 3D Printed Sodiophilic Reduced Graphene Oxide/Diamane Microlattice Aerogel for Enhanced Sodium Metal Battery Anodes
Source: Adv Sci (Weinh). 2025 Mar 31;12(22):2417638. doi: 10.1002/advs.202417638 (PMC12165111; doi:10.1002/advs.202417638)
Supplement: Supplementary file 1 — Supporting Information [file ADVS-12-2417638-s002.docx]

**Supporting Information**

**3D Printed Sodiophilic Reduced Graphene Oxide/Diamane Microlattice Aerogel for Enhanced Sodium Metal Battery Anodes**

Mengmeng Liu, Dezhi Kong, Ningning Chu, Gang Zhi, Hui Wang, Tingting Xu, Xinchang Wang, Xinjian Li, Zhuangfei Zhang,* Hui Ying Yang,* Ye Wang*

Mr. M. Liu, Dr. D. Kong, Mr. N. Chu, Mr. G. Zhi, Dr. H. Wang, Dr. T. Xu, Prof. X. Wang, Prof. X. Li, Prof. Z. Zhang, Prof. Y. Wang

Key Laboratory of Material Physics, Ministry of Education, School of Physics, Zhengzhou University, Zhengzhou 450052, China

Email: zhangzf@zzu.edu.cn; wangye@zzu.edu.cn

Prof. H. Y. Yang

Pillar of Engineering Product Development, Singapore University of Technology and Design, 8 Somapah Road, 487372, Singapore

E-mail: [yanghuiying@sutd.edu.sg](mailto:yanghuiying@sutd.edu.sg)

**Keywords:** sodium metal anode, diamane, 3D printing, sodiophilicity, dendrite-free morphology.

**Experimental Section**

*Preparation of GO, GO/diamane and NVP@C-GO ink:* GO ink was prepared via a high-speed centrifugation process. Specifically, a 30 mL dispersion of GO (purchased from Suzhou TANFENG graphene Tech Co., Ltd.) (5 mg mL^-1^) was subjected to centrifugation at 20,000 rpm for 25 min. The synthesis processes of diamane nanoflakes can be found in our previous report.^[1]^ For the subsequent preparation of the GO/diamane ink, various quantities of diamane (ranging from 16.7 to 150 mg) were introduced into the 30 mL GO solution (5 mg mL^-1^) and stirred for 12 h. Subsequently, the mixture underwent centrifugation at 20,000 rpm for 25 min to yield the GO/diamane ink suitable for 3D printing process. The protocol for the fabrication of the NVP@C cathode material has been extensively described in our previous work.^[2]^ The NVP@C-GO ink was prepared by mixing the NVP@C nanocomposites (150 mg) with the GO solution (5 mg mL^-1^, 30 mL) for 1 h, followed by centrifugation at 20,000 rpm for 25 min. To attain a homogenous printable ink, the resultant product was further ground using a quartz mortar for 30 min.

*Preparation of 3DP rGO, rGO/diamane and NVP@C-rGO microlattice aerogels:* The prepared ink was subsequently loaded into a 5 mL syringe, which was attached to the DIW 3D printer (MUSASHI, SM200SX-3A). Utilizing a dispensing nozzle with an inner diameter of 200 *μm*, the ink was extruded to form the designed 3D microlattice network pattern onto a glass substrate. During the printing process, the applied pressure was carefully controlled within the range of 0.15 and 0.25 MPa, while the syringe moved at a speed of 5 mm s^-1^. Subsequently, the 3D microlattice network on the glass substrate was subjected to freeze-drying in a freeze-dryer at -40 ℃ for 48 h to eliminate residual moisture. Ultimately, the 3DP rGO, rGO/diamane and NVP@C-rGO microlattice aerogels were achieved through thermal annealing the 3DP GO, GO/diamane and NVP@C-GO aerogel in Ar gas at 450 ℃ for a duration of 2 h.

*Fabrication of 3DP Na@rGO and 3DP Na@rGO/diamane:* The fabrication of the Na@rGO and Na@rGO/diamane was achieved via a molten-diffusion process. Specifically, within an argon-filled glovebox to maintain an inert atmosphere, metallic Na was initially heated to a temperature of 400 ℃. Subsequently, the 3DP rGO and 3DP rGO/diamane were respectively immersed into the molten Na, leading to the formation of the 3DP Na@rGO and 3DP Na@rGO/diamane.

*Material Characterization:* The morphology of the electrodes was characterized by SEM (JEOL, JSM-6700F, Japan) and TEM (JEOL, JEM-2100, Japan). The thickness of the diamane nanoflakes was measured by AFM (Veeco nanoscope multimode II-D). The crystal structures of the diamane nanoflakes and rGO sheets were measured by XRD (Rigaku Ultima IV). The surface functional groups of the diamane nanoflakes were characterized by FTIR (Bruker VERTEX 70V). The specific surface area and pore size distribution were evaluated by N_2_ adsorption/desorption isotherm measurements (ASAP 2420, Micromeritics). XPS analysis was conducted using a Thermo Scientific Escalab 250Xi spectrometer. Raman spectroscopy tests were performed on a Horiba HR Raman Evolution spectrometer with an excitation wavelength of 532 nm laser.

*In-situ optical microscope measurement:* In-situ optical microscopy observation at room temperature and 60 ℃ was carried out using a visualization electrochemical cell (Tianjin Aida Hengsheng Technology Development Co., Ltd.) and a temperature-controlled cell (LIB-MS, Beijing Scistar Technology Co., Ltd.), respectively. The optical images were taken by an optical microscope (Cewei, LW750LJT). The cell was consisted of the working electrode (Na foil, 3DP Na@rGO, and 3DP Na@rGO/diamane), counter electrode (Na foil), and a piece of PTFE separator with a thickness of 1.0 mm. The electrolyte is 1 M NaPF_6_ in diglyme. After covering the top with a piece of transparent sapphire, the cell was finally assembled in a glovebox under an argon atmosphere with water and oxygen contents <0.1 ppm. The measured current density was set as 5 mA cm^-2^.

*Assembly of the cell and electrochemical performance evaluation:* CR2032 type coin cells were assembled in an Ar-filled glovebox with O_2_/H_2_O contents maintained below 0.1 ppm. The CEs of the electrodes were evaluated based on the asymmetric cell with the bare Na foil used as the counter electrode, and various hosts (Cu foil, 3DP rGO or 3DP rGO/diamane) as the working electrodes. The cut-off voltage, current density and the areal capacity were 0.5 V, 2 mA cm^-2^ and 1 mAh cm^-2^, respectively. The rate capability and long cycle performance was evaluated based on the symmetric cells. To control the variables, Na foil with dimensions of 1 cm × 1 cm × 0.2 cm, 3DP Na@rGO, or 3DP Na@rGO/diamane was used as both the working and counter electrodes, and filled with 1 M NaPF_6_ in diglyme electrolyte. The cut-off voltage was set as 0.5 V. The electrochemical measurement at different temperatures was conducted by putting the cell in a low/high temperature test chamber (Hyxian HZ-2019A). By setting the program, the temperature chamber was maintained at each temperature for 20 h, corresponding to 10 cycles. Then, the heating time from one temperature to the next one was less than 10 min controlled by the machine automatically. Full cells were assembled using different types of anodes (Na foil with a thickness of 2 mm, 3DP Na@rGO and 3DP Na@rGO/diamane) and an NVP cathode with 1 M NaPF_6_ in diglyme. PP separator (Celgard 2500) was used in all cells.

Galvanostatic measurements of the electrodes were conducted utilizing a Neware multichannel battery tester at room temperature. EIS and Tafel assessments were performed on a Biologic VMP3 electrochemical workstation. The performance of the full cell was evaluated within a potential window of 2.0 to 3.8 V. The specific capacity of the full cell was calculated based on the mass of NVP@C cathode.

*COMSOL simulation:* To determine the surface current density distribution of Na, Na@rGO and Na@rGO/diamane, COMSOL Multiphysics with the physics module of “secondary current distribution” was applied to perform the corresponding simulations. To simplify the simulation model of the Na@rGO/diamane, rGO nanoflakes were handled as a sheet with a width of 1 *μm* and a length of 5 *μm*, respectively. The diameter of the diamane is set as 500 nm, and the protrusions on the Na surface are set with a width of 20 nm and a height of 30 nm.

*Theoretical calculation:* The binding energy between sodium and diamane, as well as graphene, was calculated via Density Functional Theory (DFT) implemented within the Vienna Ab Initio Simulation Package (VASP).^[3]^ The Generalized Gradient Approximation (GGA) was utilized to delineate the exchange-correlation interactions of the itinerant electrons, with the Perdew-Burke-Ernzerhof (PBE) functional serving as the descriptor for these interactions.^[4]^ A kinetic energy cut-off of 600 eV was imposed across all computational tasks, complemented by a k-point mesh configuration of 7×7×1. The convergence thresholds were delineated as 10^-5^ eV per atom for energy and 10^-3^ eV Å^-1^ for force per atom. To mitigate spurious interactions attributable to the periodic boundary conditions, a vacuum layer of 15 Å was deliberately selected for the slab calculations.

**Supporting Figures and Tables**


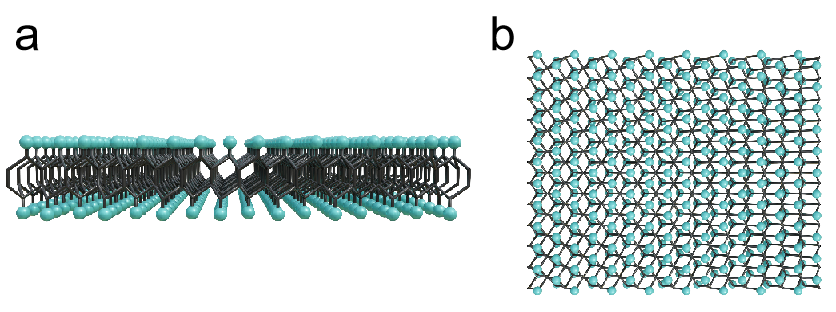


**Figure S1.** a) Front and b) top views of the schematic diagram of diamane.


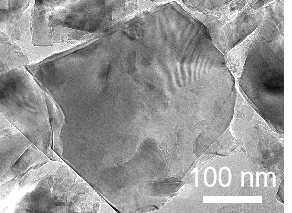


**Figure S2.** TEM image of diamane nanoflakes.


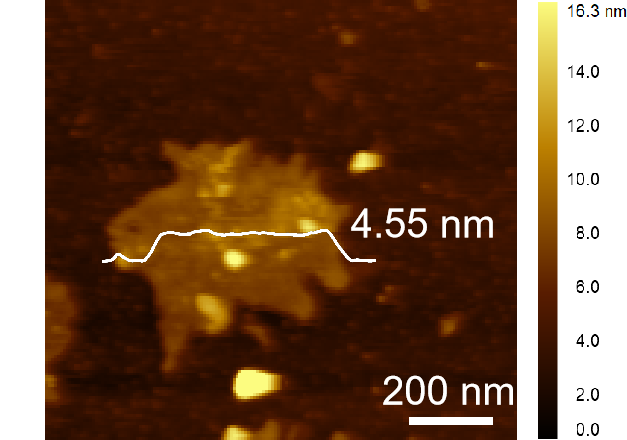


**Figure S3.** AFM image of diamane nanoflakes.


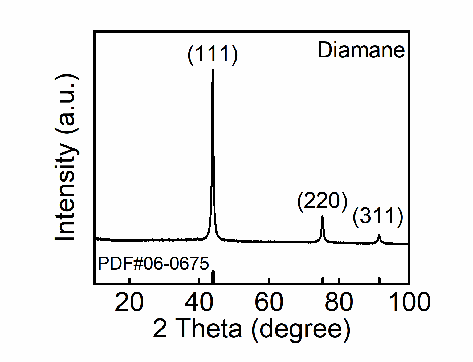


**Figure S4.** XRD pattern of diamane nanoflakes.


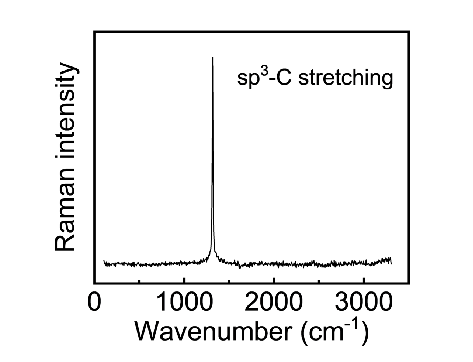


**Figure S5.** Raman spectrum of diamane nanoflakes.


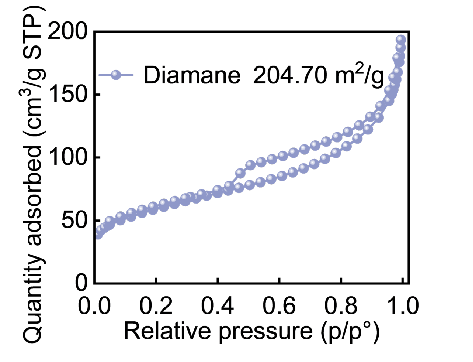


**Figure S6.** The N_2_ adsorption/desorption isotherms of diamane nanoflakes.


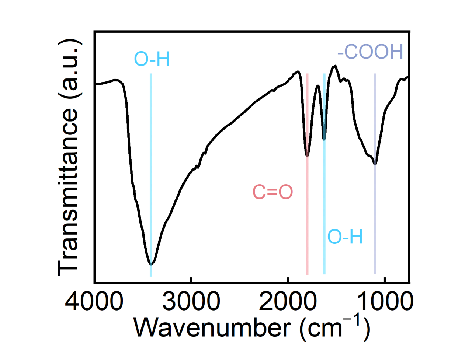


**Figure S7.** FTIR spectrum of diamane nanoflakes.


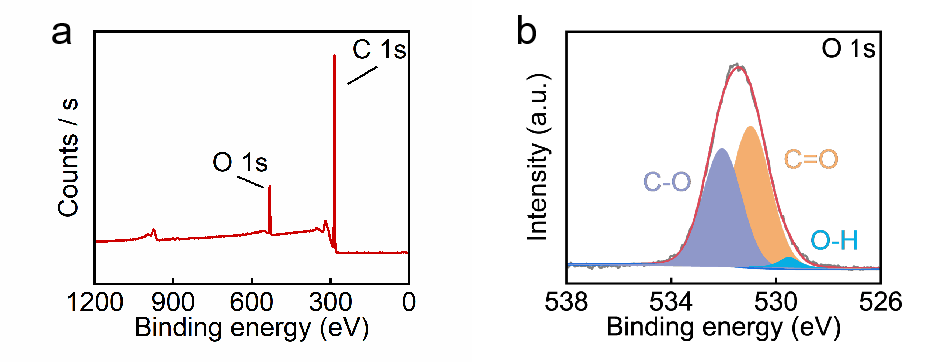


**Figure S8.** a) XPS survey spectrum of diamane and b) corresponding high-resolution XPS spectra of O 1s.


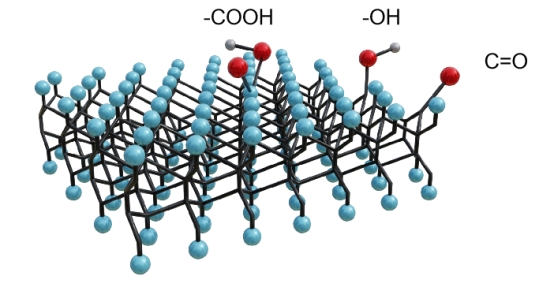


**Figure S9.** Schematic diagram of oxygen-containing functional groups on the surface of diamane.


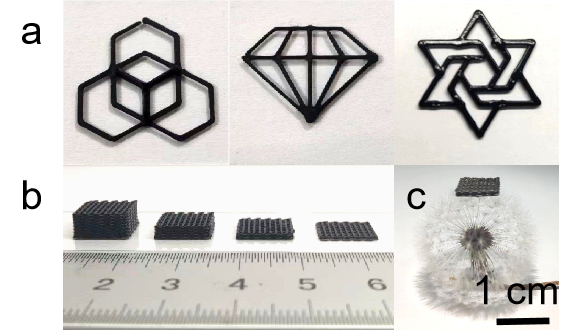


**Figure S10.** a) Various patterns, b) thicknesses printed by DIW 3D printing and c) an optical photo of a 3D printed rGO/diamane electrode on a dandelion flower to show its light weight property.


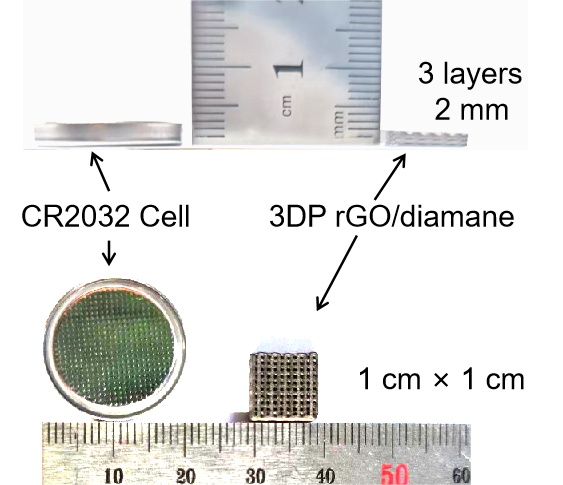


**Figure S11.** Height and size of a 3DP rGO/diamane electrode with 3 layers (one vertical and one horizontal layer counted as one layer).


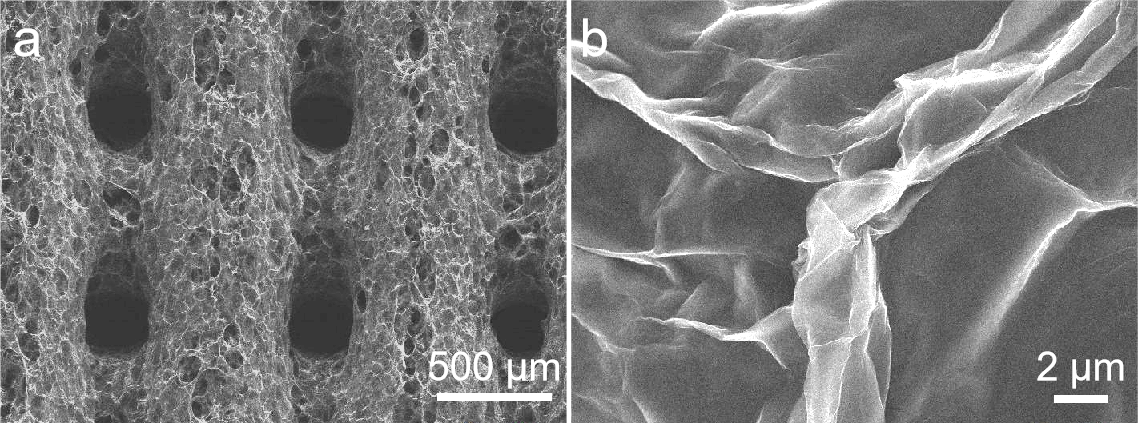


**Figure S12.** SEM images of 3DP rGO microlattice aerogel with various magnifications.


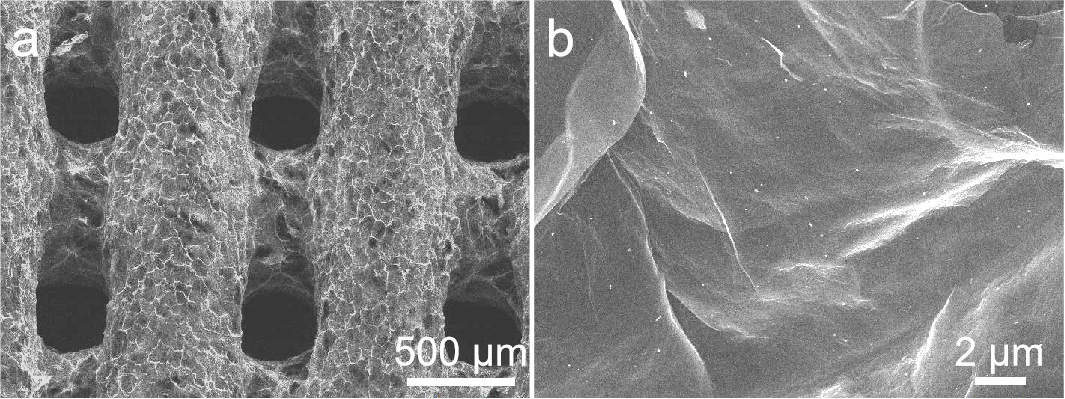


**Figure S13.** SEM images of 3DP rGO/diamane-10 microlattice aerogel with various magnifications.


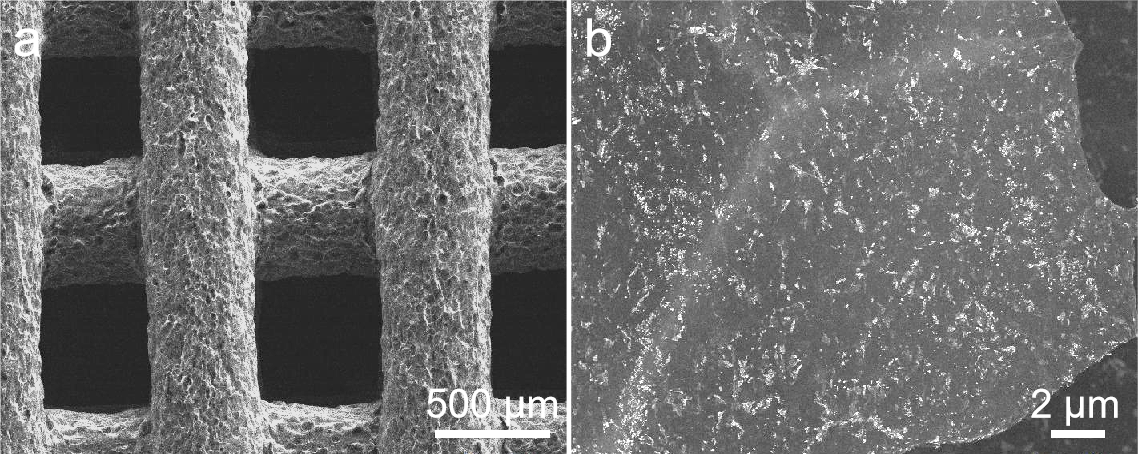


**Figure S14.** SEM images of 3DP rGO/diamane-50 microlattice aerogel with various magnifications.


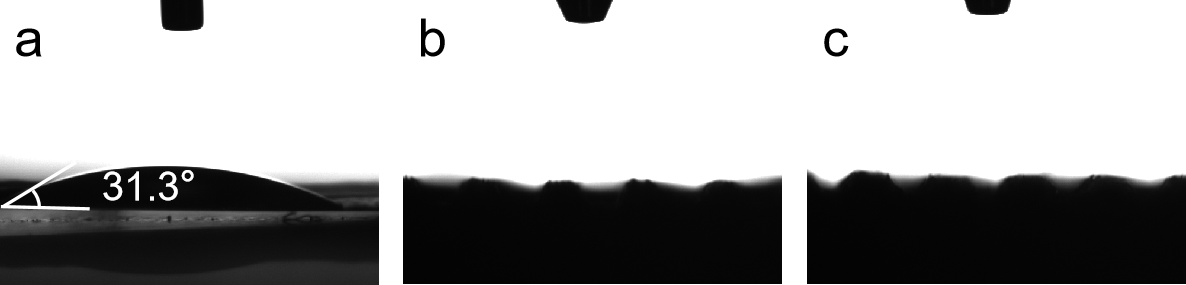


**Figure S15.** Contact angles between electrolyte and a) Cu, b) 3DP rGO, and c) rGO/diamane electrodes.


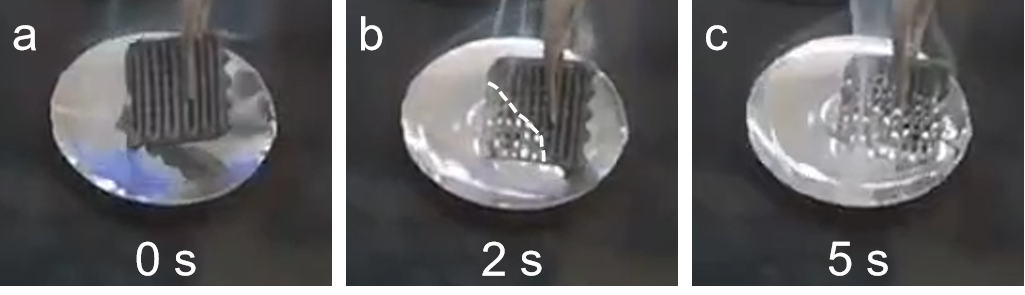


**Figure S16.** Time-lapse images of the molten Na diffused into the 3DP rGO/diamane host at a) 0 s, b) 2 s and c) 5 s.


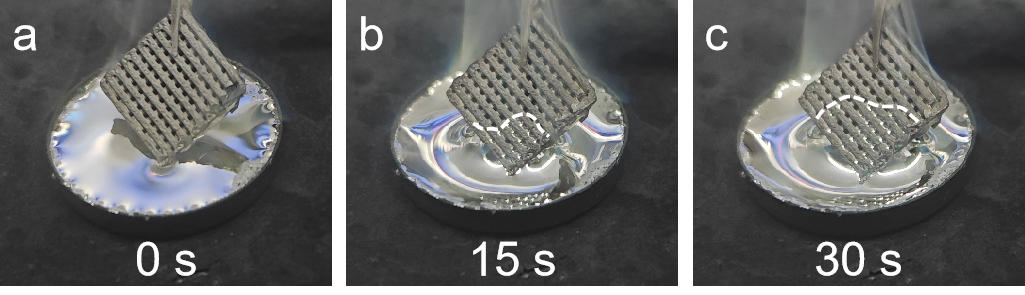


**Figure S17.** Time-lapse images of the molten Na diffused into the 3DP rGO host at a) 0 s, b) 15 s and c) 30 s.


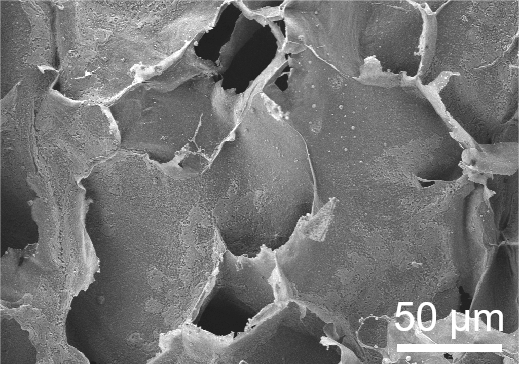


**Figure S18.** SEM image of Na@rGO/diamane electrode by infusing the molten Na into the rGO/diamane microlattice aerogel.


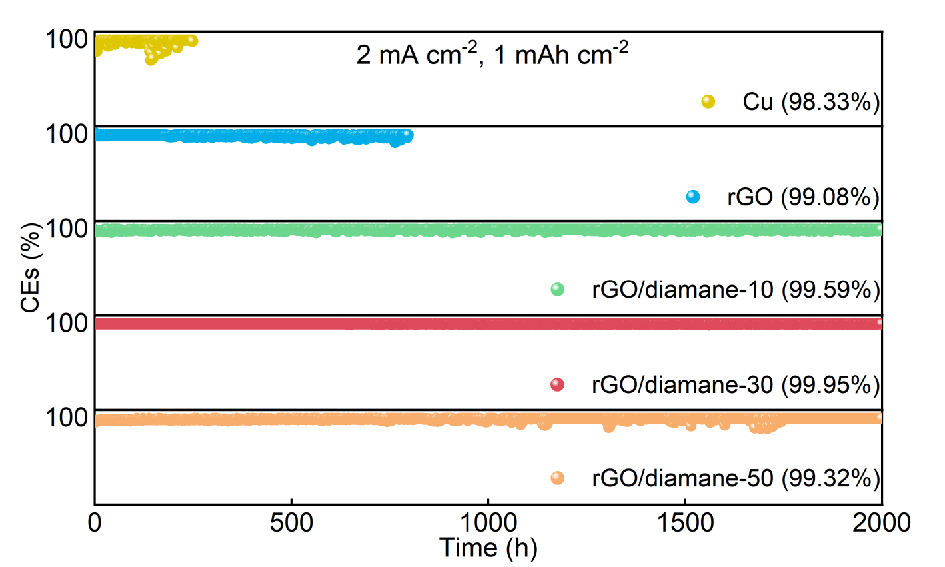


**Figure S19.** CEs of various electrodes at 2 mA cm^-2^ with 1 mAh cm^-2^.


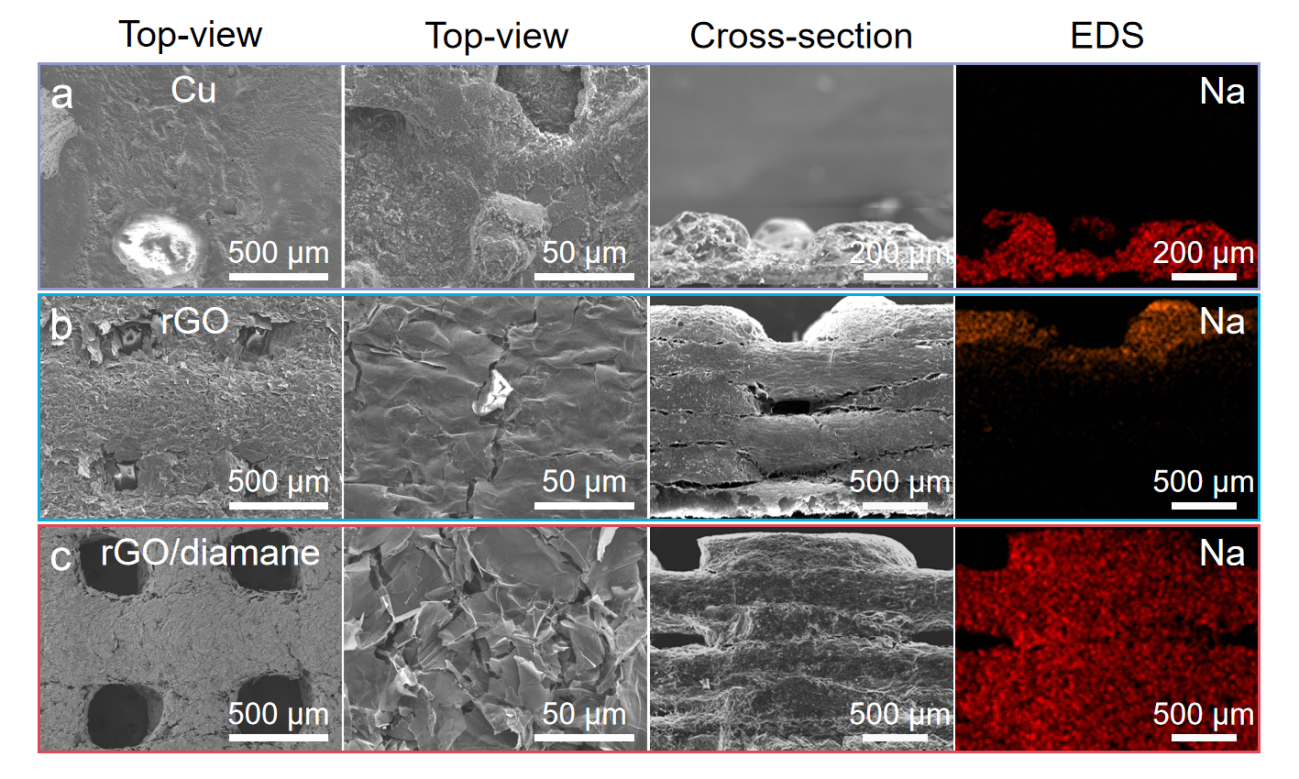


**Figure S20.** Ex-situ SEM, cross-sectional images and corresponding EDS Na elemental mapping of the a) Cu, b) 3DP rGO, and c) 3DP rGO/diamane microlattice electrodes.


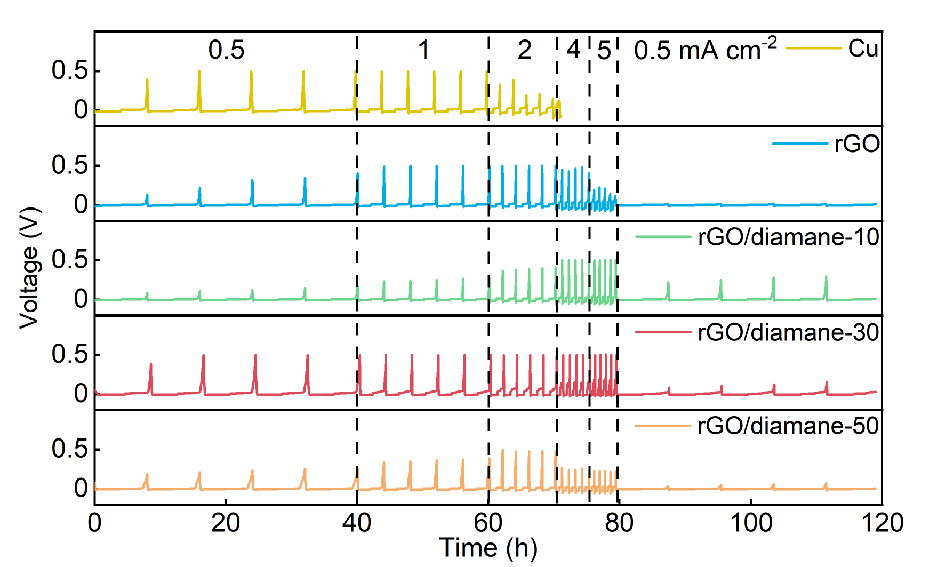


**Figure S21.** The rate performance of various electrodes at different current densities based on asymmetric cell.


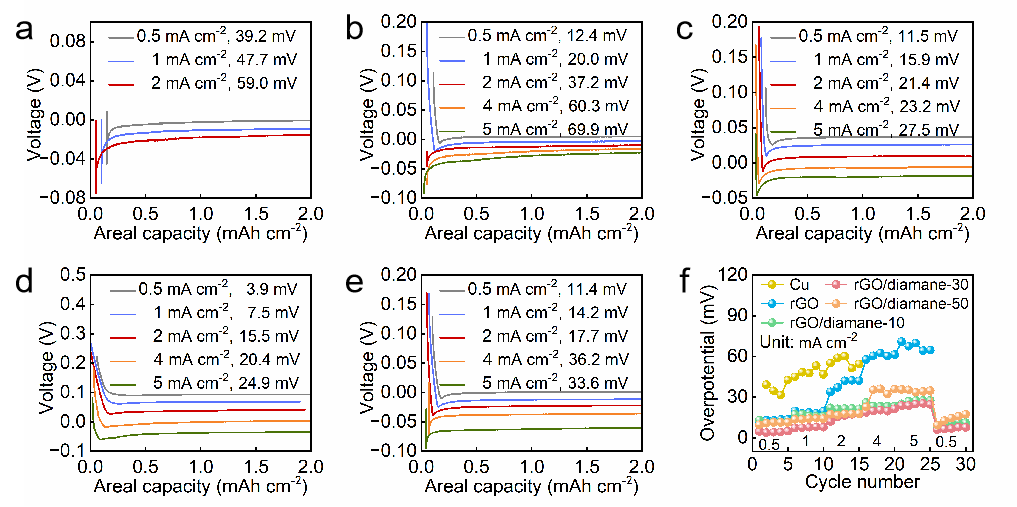


**Figure S22.** Voltage profiles of the a) Cu, b) 3DP rGO, c) 3DP rGO/diamane-10, d) 3DP rGO/diamane-30 and e) 3DP rGO/diamane-50 electrodes, and f) the nucleation overpotentials at various current densities.


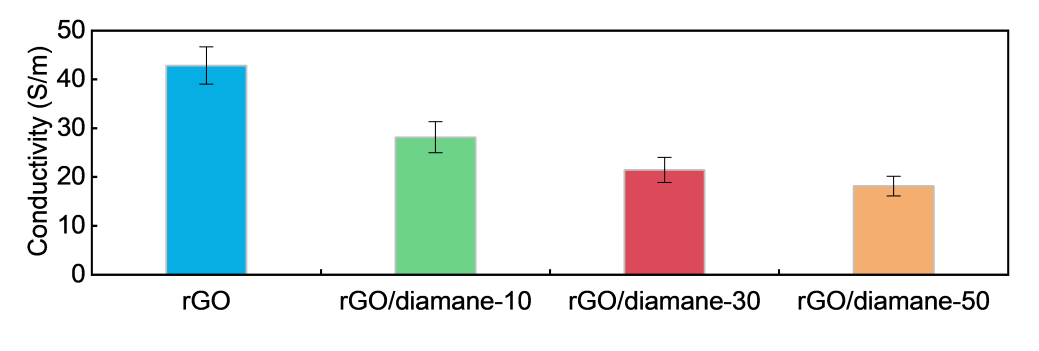


**Figure S23.** The conductivity of electrodes with various quantities of diamane.


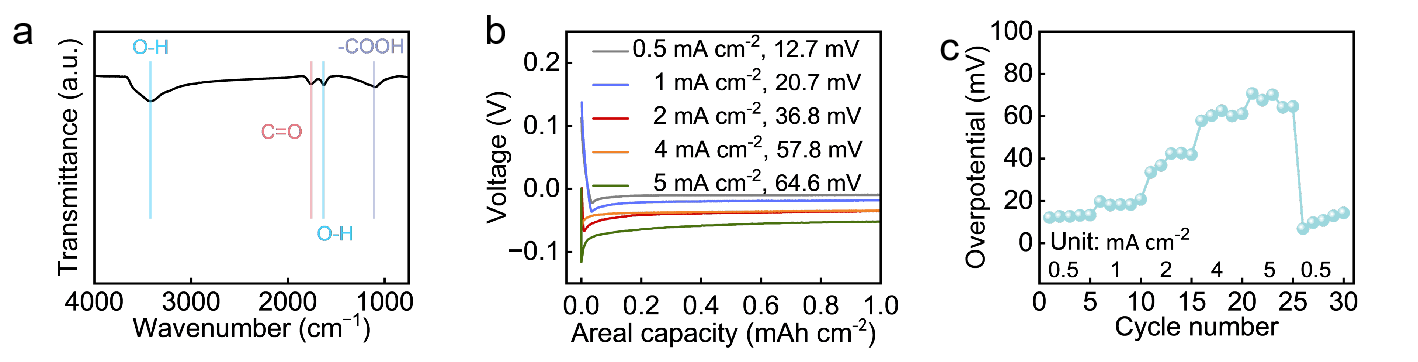


**Figure S24.** a) FTIR spectrum of 3DP rGO/diamane-30 after removing surface functional groups by annealing in Ar/H_2_ atmosphere. b) Voltage profiles of the 3DP rGO/diamane after removing surface functional groups of diamane by annealing in Ar/H_2_ atmosphere, and c) the nucleation overpotential at various current densities.


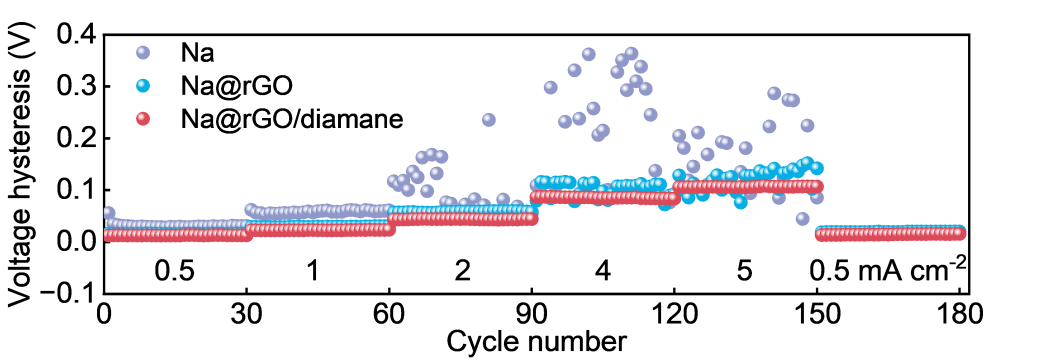


**Figure S25.** The voltage hysteresis of Na, 3DP Na@rGO and Na@rGO/diamane electrodes at 0.5, 1, 2, 4 and 5 mA cm^-2^.


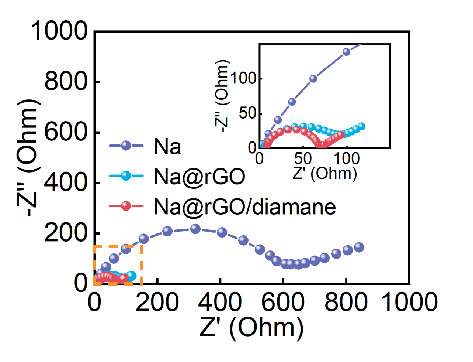


**Figure S26.** EIS curves of Na, 3DP Na@rGO and Na@rGO/diamane anodes before cycle. The inset is the enlarged image of the curves in the dashed square.

The EIS equivalent circuit is shown in the insert of Figure 3f, where R_s_ is the electrolyte resistance, R_f_ is the SEI layer resistance, CPE_1_ and CPE_2_ are constant phase elements for SEI layer and double layer, respectively; and W_1_ is the Warburg impedance related to the straight line in the low-frequency region related to the sodium diffusion process.^[5]^


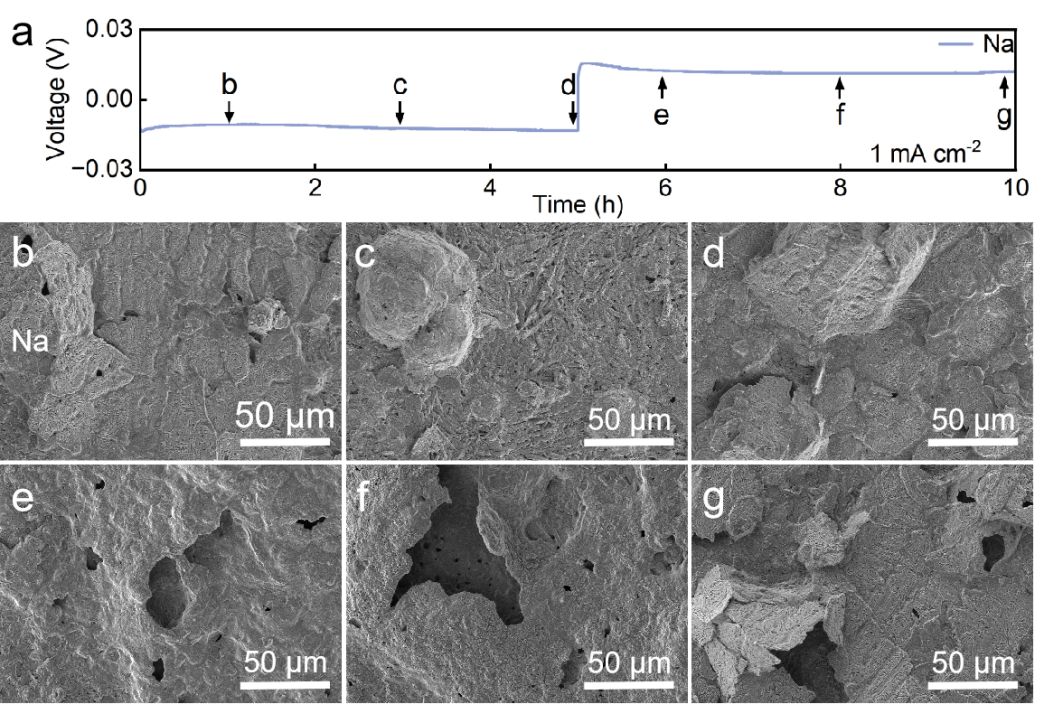


**Figure S27.** a) Voltage profile of the pure Na anode at 1 mA cm^-2^ with 5 mAh cm^-2^. SEM images of the pure Na with deposition capacity of b) 1, c) 3, and d) 5 mAh cm^-2^ and stripping capacity of e) 1, f) 3 and g) 5 mAh cm^-2^ at 1 mA cm^-2^.


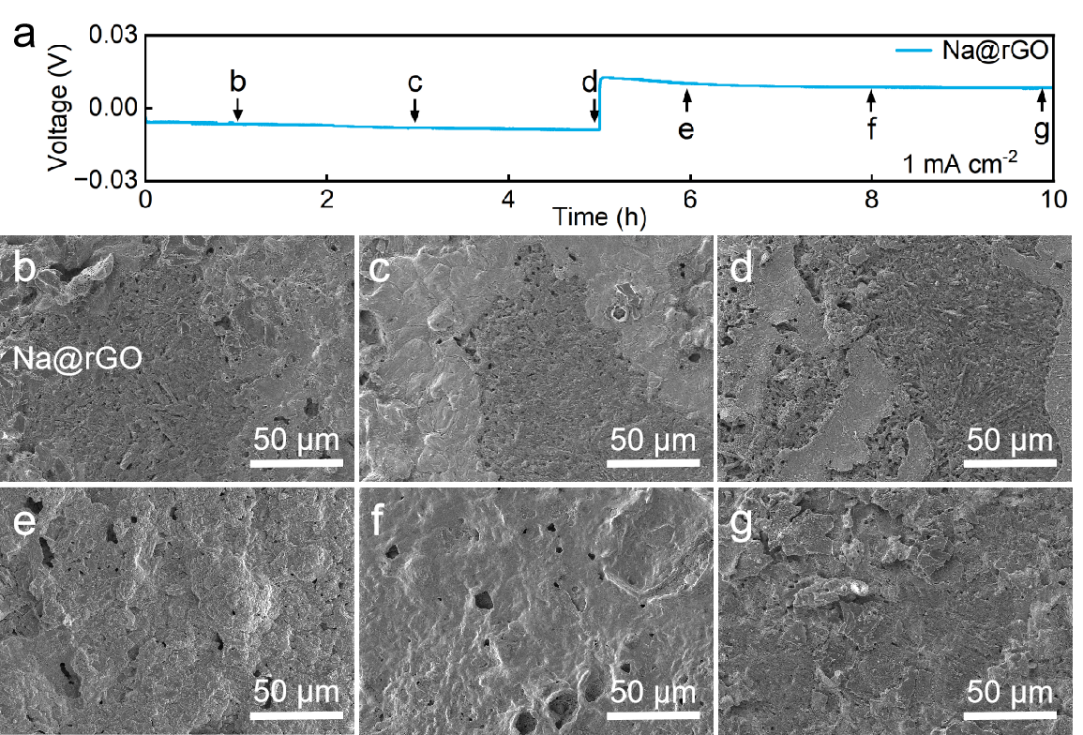


**Figure S28.** a) Voltage profile of the Na@rGO anode at 1 mA cm^-2^ with 5 mAh cm^-2^. SEM images of the Na@rGO with deposition capacity of b) 1, c) 3, and d) 5 mAh cm^-2^ and stripping capacity of e) 1, f) 3 and g) 5 mAh cm^-2^ at 1 mA cm^-2^.


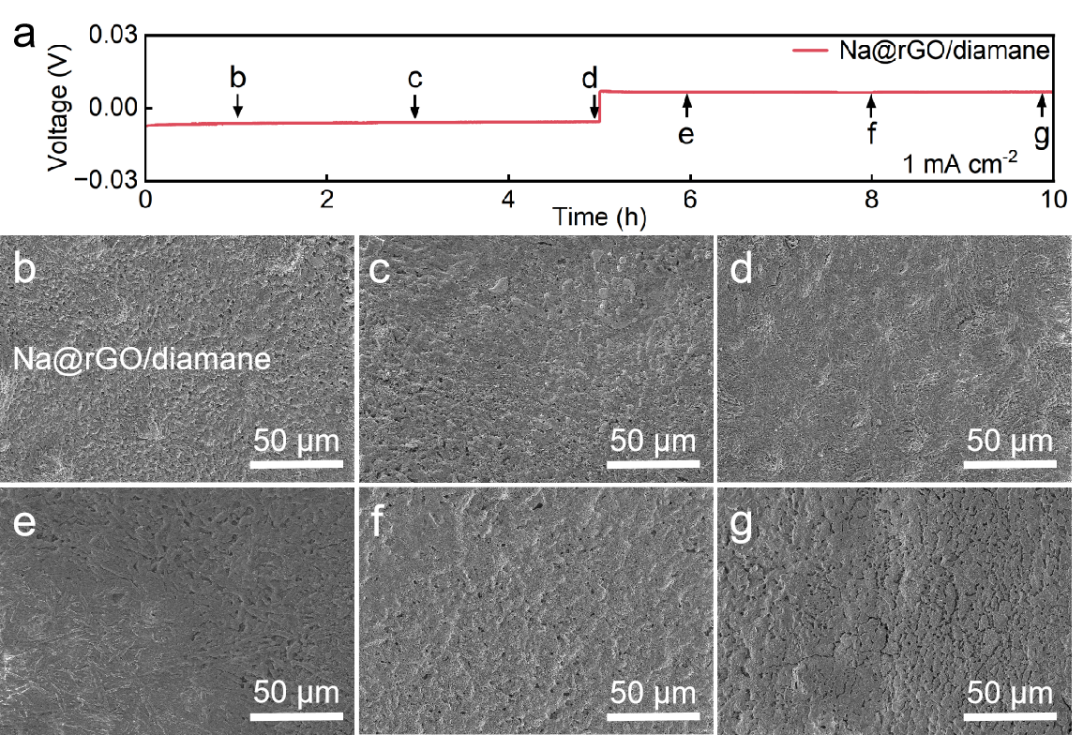


**Figure S29.** a) Voltage profile of the Na@rGO/diamane anode at 1 mA cm^-2^ with 5 mAh cm^-2^. SEM images of the Na@rGO/diamane with deposition capacity of b) 1, c) 3, and d) 5 mAh cm^-2^ and stripping capacity of e) 1, f) 3 and g) 5 mAh cm^-2^ at 1 mA cm^-2^.


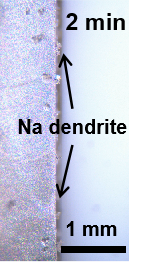


**Figure S30.** In-situ optical microscopy image of Na dendrites nucleated on the surface of Na anode at 5 mA cm^-2^ for 2 min.


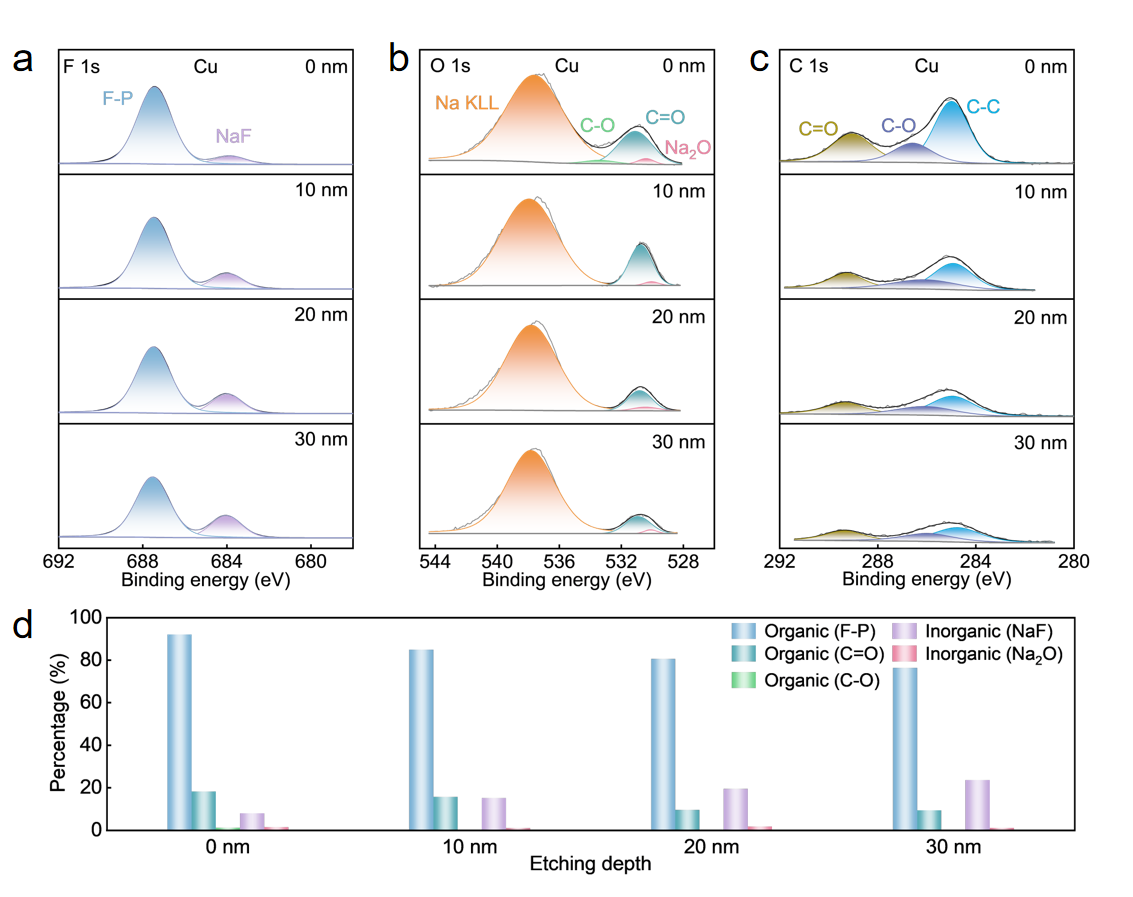


**Figure S31.** XPS depth profiles of a) F 1s, b) O 1s, and c) C 1s of Cu foil electrode after 10 cycles at 2 mA cm^-2^ with 1 mAh cm^-2^. d) The content comparison of the organic and inorganic species at different depths.


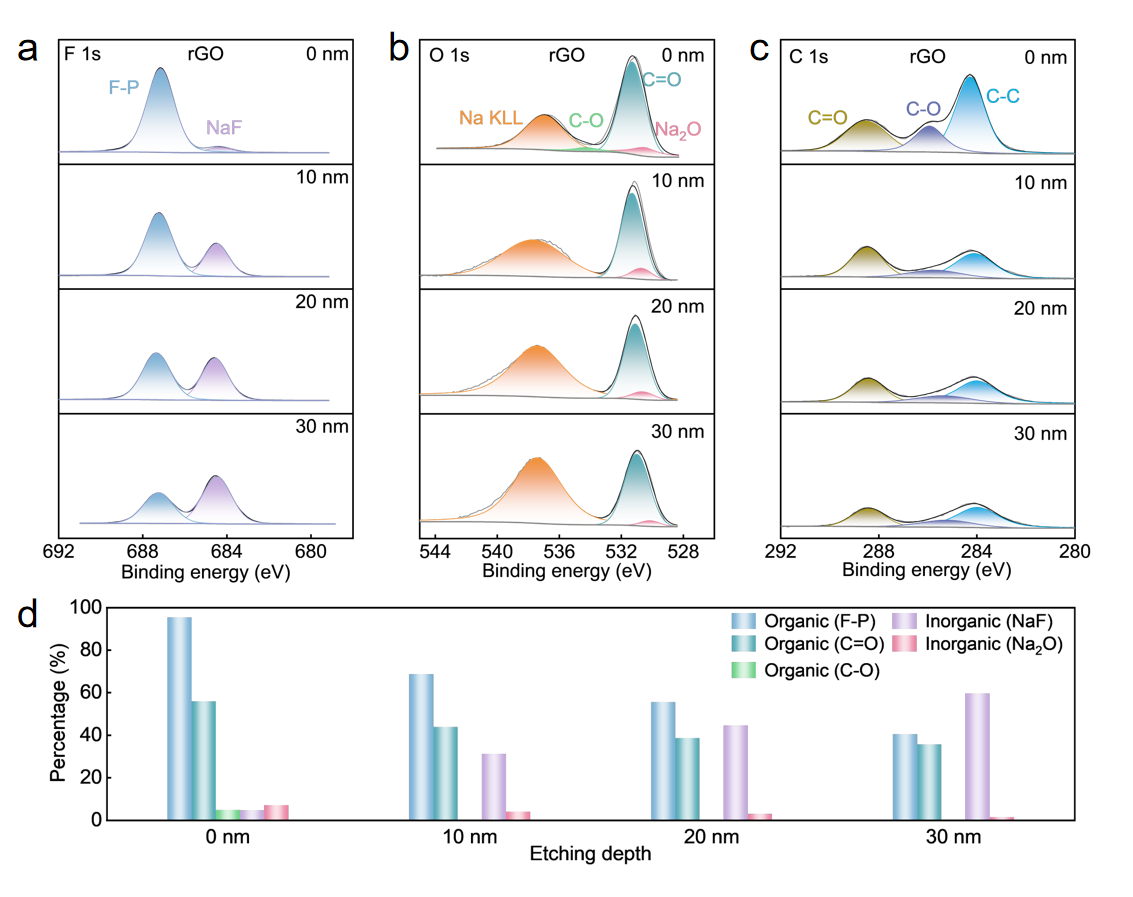


**Figure S32.** XPS depth profiles of a) F 1s, b) O 1s, and c) C 1s of 3DP rGO electrode after 10 cycles at 2 mA cm^-2^ with 1 mAh cm^-2^. d) The content comparison of the organic and inorganic species at different depths.


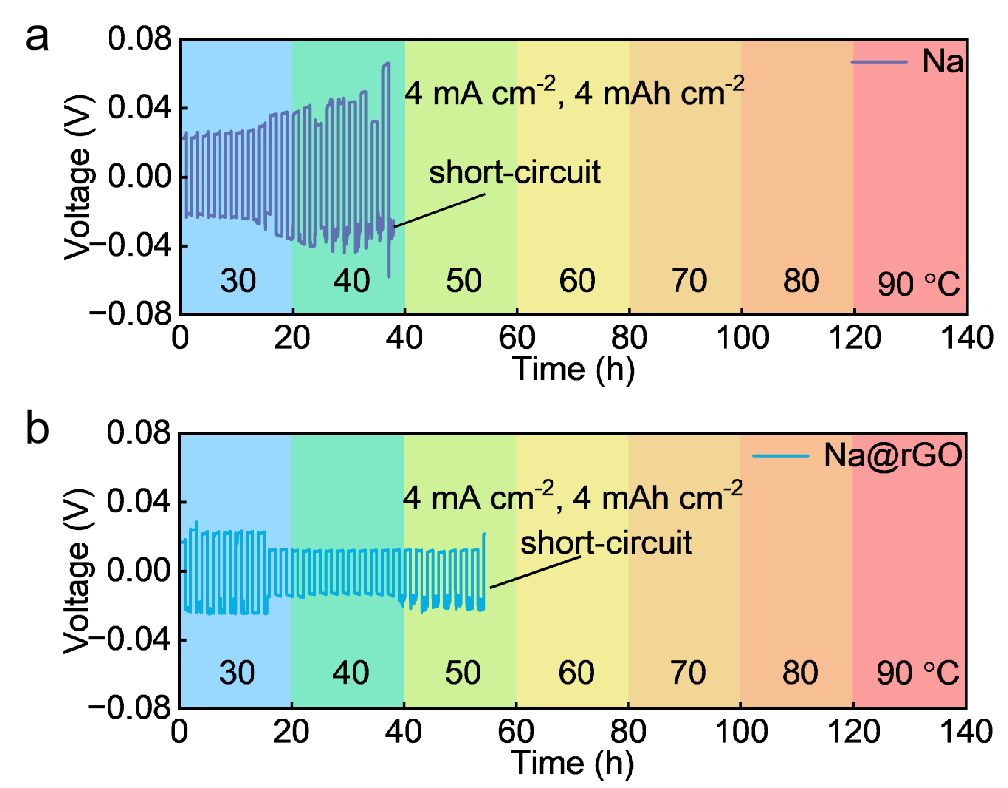


**Figure S33.** GCD voltage curves of a) Na and b) 3DP Na@rGO symmetric cells at 4 mA cm^-2^ with 4 mAh cm^-2^ at different temperatures.


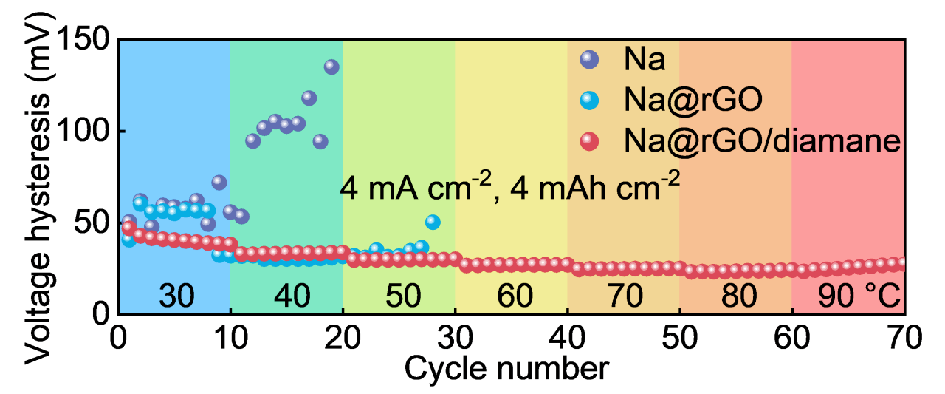


**Figure S34.** The voltage hysteresis of the Na, 3DP Na@rGO and Na@rGO/diamane electrodes at 4 mA cm^-2^ with 4 mAh cm^-2^ at different temperatures.


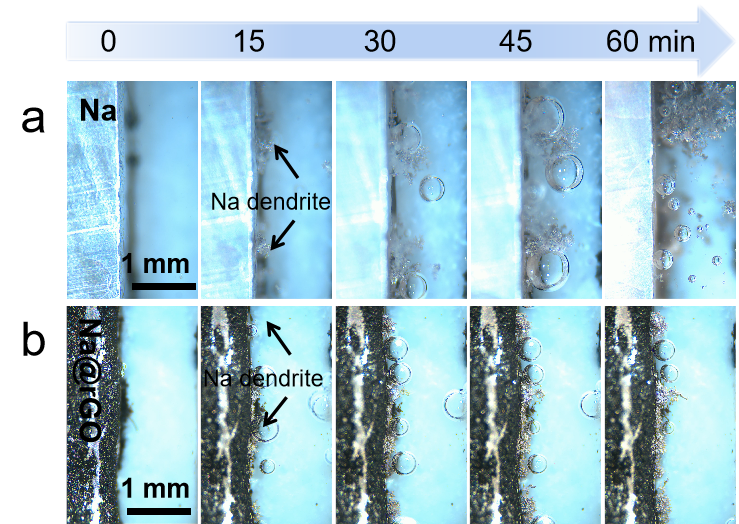


**Figure S35.** a) Na and b) 3DP Na@rGO deposition morphology evolution measured by in-situ optical microscopy at 5 mA cm^-2^ for 1 h at 60 ℃.


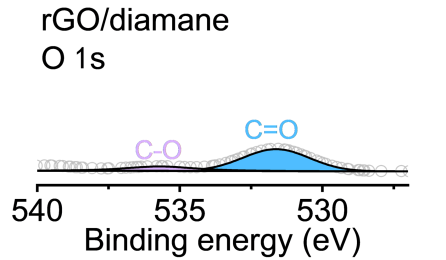


**Figure S36.** High-resolution XPS: O 1s profiles of the 3DP rGO/diamane electrode after 10 cycles at 2 mA cm^-2^ with 1 mAh cm^-2^ at 60 ℃.


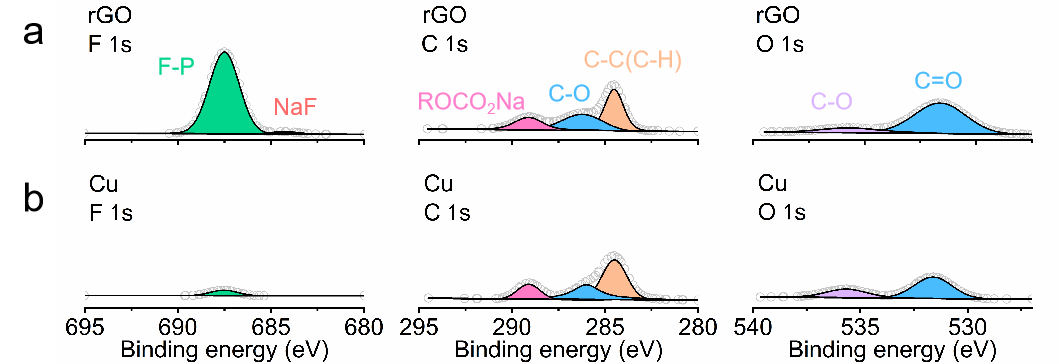


**Figure S37.** High-resolution XPS: F 1s, C 1s and O 1s profiles of the a) 3DP rGO, and b) Cu electrodes after 10 cycles at 2 mA cm^-2^ with 1 mAh cm^-2^ at 60 ℃.


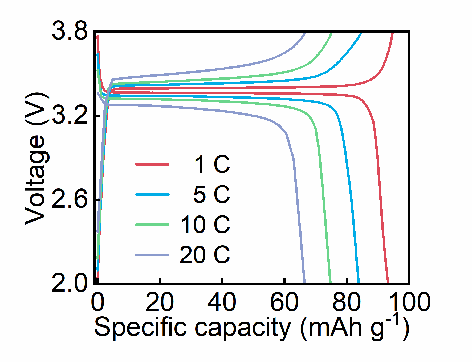


**Figure S38.** GCD curves of the 3DP NVP@C-rGO||3DP Na@rGO/diamane full cell at various current densities.


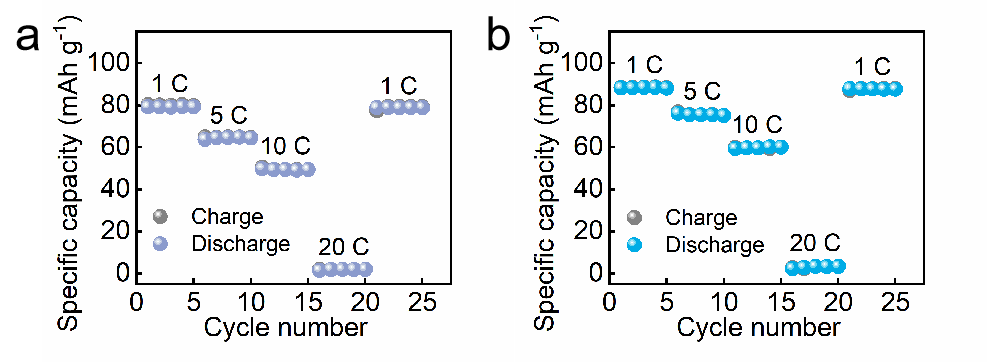


**Figure S39.** Rate capability of a) 3DP NVP@C-rGO||Na and b) 3DP NVP@C-rGO||3DP Na@rGO full cells.


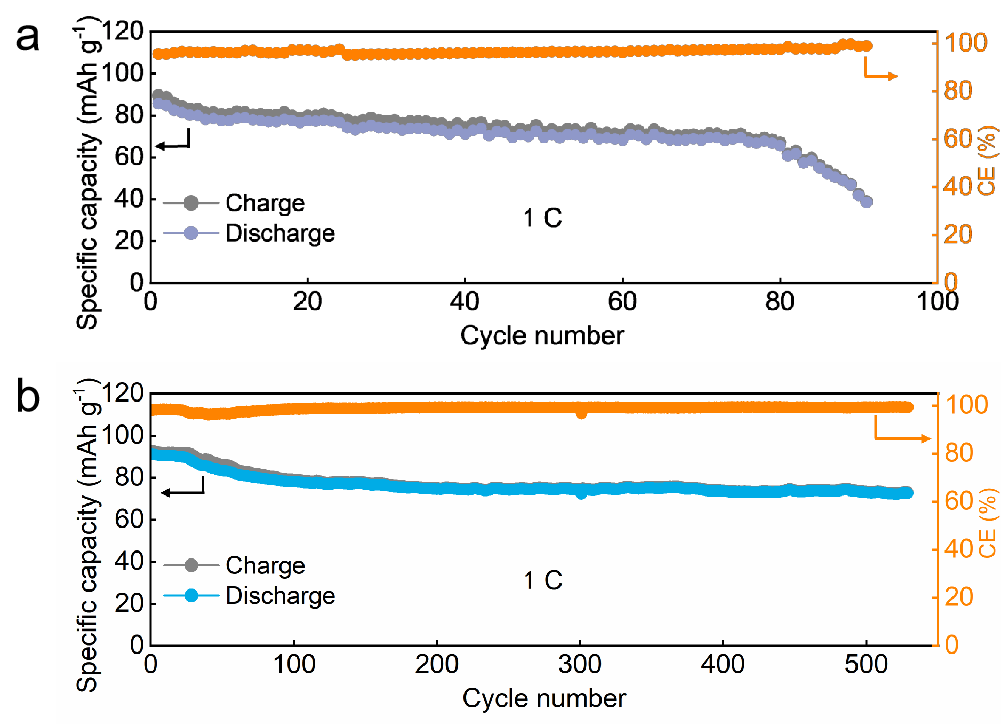


**Figure S40.** Cycling performance and related CEs of the full cells with a) Na foil and b) 3DP Na@rGO anodes at 1 C.

###


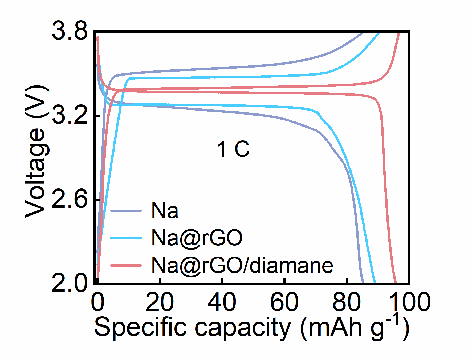


**Figure S41.** GCD curves of the full cells with Na, 3DP Na@rGO and Na@rGO/diamane anodes at 1 C.


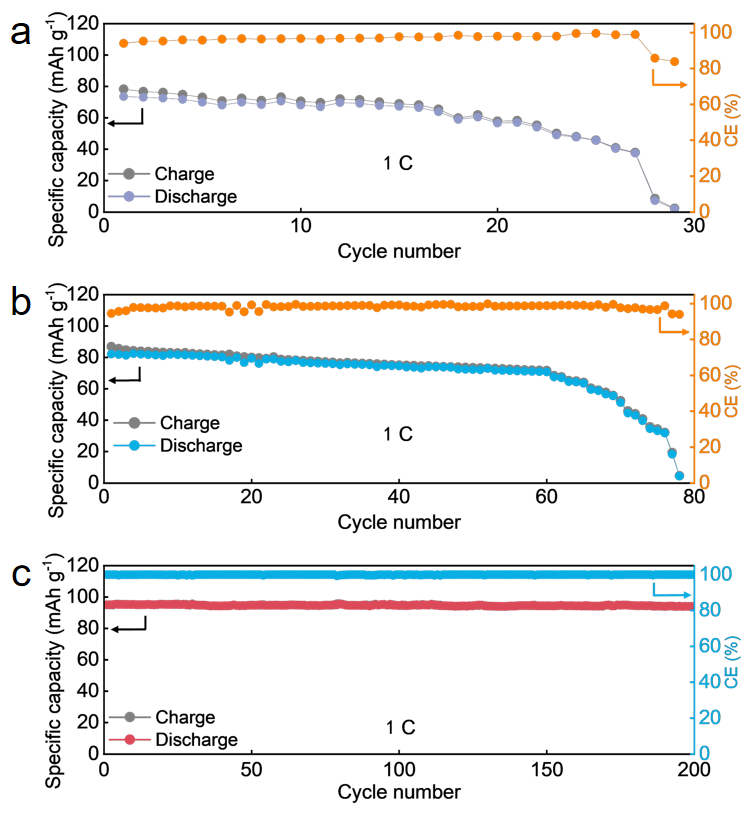


**Figure S42.** Cycling performance and related CE of the full cells with (a) Na@Cu, (b) Na@rGO and (c) Na@rGO/diamane anodes with the N/P ratio of 1.8 at 1 C.

**Table S1.** Comparison of electrochemical performance of carbon-based nanomaterials for Na metal anodes.

| **Electrode** | **Current density**  **(mA cm^-2^)** | **Capacity**  **(mAh cm^-2^)** | **Cycle time (h)** | **Reference** |
| --- | --- | --- | --- | --- |
| **oxygen-containing carbonized coconut framework** | 5 | 10 | 4000 | ^[6]^ |
| **3D printed rGO/CNT** | 2 | 1 | 1700 | ^[7]^ |
| **Skeletal CNF** | 3 | 3 | 1000 | ^[8]^ |
| **Gradiently graphitized 3D CF** | 1 | 5 | 1500 | ^[9]^ |
| **3D printed graphene lattice** | 1 | 1 | 1000 | ^[10]^ |
| **Graphitized carbon microsphere** | 0.5 | 2 | 3500 | ^[11]^ |
| **Porous RGO** | 1 | 1 | 2000 | ^[12]^ |
| **Holey carbon nanosheet** | 1 | 1 | 2000 | ^[13]^ |
| **3D carbon felt** | 1 | 2 | 500 | ^[14]^ |
| **vertical graphene sheets on CF** | 1 | 1 | 3200 | ^[15]^ |
| **N-functionalized hard carbon** | 1 | 1 | 1700 | ^[16]^ |
| **oxygen-doped CNT framework** | 0.5 | 1 | 4000 | ^[17]^ |
| **rGO aerogel** | 5 | 1 | 400 | ^[18]^ |
| **CNT-Na composite** | 0.5 | 1 | 800 | ^[19]^ |
| **3DP rGO/diamane** | 1 | 1 | 7200 | **This work** |

**Table S2.** *R*_ct_ of the Na, 3DP Na@rGO, Na@rGO/diamane-10, 30 and 50 electrodes before and after 10 cycles, and the diffusion coefficients (*D*) of the above electrodes after 10 cycles.

| **Electrode** | ***R*_ct_ (Ω)**  **Before cycles** | ***R*_ct_ (Ω)**  **After 10 cycles** | **σ** | ***D***  **(cm^2^ s^-1^)** |
| --- | --- | --- | --- | --- |
| **Na** | 580.30 | 104.80 | 50.67 | 3.82×10^-16^ |
| **Na@rGO** | 75.72 | 71.19 | 20.75 | 2.28×10^-15^ |
| **Na@rGO/diamane** | 56.68 | 29.64 | 6.15 | 2.60×10^-14^ |

**Table S3**. Comparison of rate capability of the full cells with Na foil, 3DP Na@rGO and Na@rGO/diamane anodes.

| **Electrode** | Capacity (mAh g^-1^) at 1 C | Capacity (mAh g^-1^) at 5 C | Capacity (mAh g^-1^) at 10 C | Capacity (mAh g^-1^) at 20 C |
| --- | --- | --- | --- | --- |
| **Na** | 79.21 | 64.38 | 49.39 | 1.77 |
| **Na@rGO** | 88.31 | 75.50 | 59.79 | 2.97 |
| **Na@rGO/diamane** | 93.15 | 83.87 | 75.17 | 66.87 |

**Table S4.** Comparison of electrochemical performance of various two-dimensional nanomaterial-based sodium metal batteries.

| **Electrode** | **Cycle number** | **Capacity (mAh g^-1^)** | **Reference** |
| --- | --- | --- | --- |
| **3DP Ag/rGO** | 500 | 80.69 | ^[20]^ |
| **3DP V_2_CT_x_/rGO-CNT** | 400 | 86.27 | ^[21]^ |
| **3DP Au/rGO** | 205 | 84.95 | ^[22]^ |
| **3DP rGO/CNT** | 100 | 67.6 | ^[7]^ |
| **3DP Ti_3_C_2_T_x_/rGO** | 500 | 85.3 | ^[23]^ |
| **3DP N-doped graphene aerogel** | 1000 | 87.5 | ^[24]^ |
| **3DP Co_3_O_4_@C/rGO** | 500 | 97.97 | ^[25]^ |
| **3DP Nb_2_CT_x_/rGO** | 200 | 90.22 | ^[26]^ |
| **N-doped carbon nanosheet** | 200 | 70.77 | ^[27]^ |
| **MXene@g-C_3_N_4_** | 800 | 88 | ^[28]^ |
| **Porous RGO** | 350 | 95 | ^[12]^ |
| **rGO aerogel** | 100 | 79 | ^[18]^ |
| **Holey carbon nanosheet** | 100 | 105.3 | ^[13]^ |
| **3DP rGO/diamane** | 2000 | 91.37 | **This work** |

**Supporting references**

[1] H. W. Aijiao Li, Xiaobing Liu, Weixia Shen, Chao Fang, Zhuangfei Zhang, Yuewen Zhang, Liangchao Chen, Qianqian Wang, Biao Wan, Ye Wang, Chongxin Shan, *Chem. Eng. J.* **2024**, *491*, 151914.

[2] S. Huang, S. Fan, L. Xie, Q. Wu, D. Kong, Y. Wang, Y. V. Lim, M. Ding, Y. Shang, S. Chen, H. Y. Yang, *Adv. Energy Mater.* **2019**, *9*, 1901584.

[3] P. E. Blochl, *Physical Review B* **1994**, *50*, 17953.

[4] J. P. Perdew, K. Burke, M. Ernzerhof, *Phsical Review Letters* **1996**, *77*, 3865.

[5] Y. Wang, Z. Han, S. Yu, R. Song, H. Song, K. K. Ostrikov, H. Yang, *Carbon* **2013**, *64*, 230.

[6] T. Li, J. Sun, S. Gao, B. Xiao, J. Cheng, Y. Zhou, X. Sun, F. Jiang, Z. Yan, S. Xiong, *Adv. Energy Mater.* **2021**, *11*, 2003699.

[7] J. Yan, G. Zhi, D. Kong, H. Wang, T. Xu, J. Zang, W. Shen, J. Xu, Y. Shi, S. Dai, X. Li, Y. Wang, *J. Mater. Chem. A* **2020**, *8*, 19843.

[8] N. Mubarak, F. Rehman, M. Ihsan‐Ul‐Haq, M. Xu, Y. Li, Y. Zhao, Z. Luo, B. Huang, J. K. Kim, *Adv. Energy Mater.* **2022**, *12*, 2103904.

[9] Z. Sun, Y. Ye, J. Zhu, E. Zhou, J. Xu, M. Liu, X. Kong, S. Jin, H. Ji, *Small* **2022**, *18*, 2107199.

[10] Y. Yu, Z. Wang, Z. Hou, W. Ta, W. Wang, X. Zhao, Q. Li, Y. Zhao, Q. Zhang, Z. Quan, *ACS Appl. Energy Mater.* **2019**, *2*, 3869.

[11] H. Ye, C.-Y. Wang, T.-T. Zuo, P.-F. Wang, Y.-X. Yin, Z.-J. Zheng, P. Wang, J. Cheng, F.-F. Cao, Y.-G. Guo, *Nano Energy* **2018**, *48*, 369.

[12] K. Yan, S. Zhao, J. Zhang, J. Safaei, X. Yu, T. Wang, S. Wang, B. Sun, G. Wang, *Nano Lett.* **2020**, *20*, 6112.

[13] Y. Xie, Z. Han, H. Li, J. Hu, L. Zhang, A. Wang, S. Chang, J. Xu, C. Liu, Y. Lai, Z. Zhang, *Chem. Eng. J.* **2022**, *427*, 130959.

[14] S. S. Chi, X. G. Qi, Y. S. Hu, L. Z. Fan, *Adv. Energy Mater.* **2018**, *8*, 1702764.

[15] X. Ji, Z. Lin, J. Zeng, Y. Lin, Y. Mu, S. Wang, Z. Ren, J. Yu, *Carbon* **2020**, *158*, 394.

[16] J. Liang, W. Wu, L. Xu, X. Wu, *Carbon* **2021**, *176*, 219.

[17] C. Chu, N. Wang, L. Li, L. Lin, F. Tian, Y. Li, J. Yang, S.-x. Dou, Y. Qian, *Energy Storage Mater.* **2019**, *23*, 137.

[18] F. Wu, J. Zhou, R. Luo, Y. Huang, Y. Mei, M. Xie, R. Chen, *Energy Storage Mater.* **2019**, *22*, 376.

[19] Y.-J. Kim, J. Lee, S. Yuk, H. Noh, H. Chu, H. Kwack, S. Kim, M.-H. Ryou, H.-T. Kim, *J. Power Sources* **2019**, *438*, 227005.

[20] Y. Liu, H. Wang, H. Yang, Z. Wang, Z. Huang, D. Pan, Z. Zhang, Z. Duan, T. Xu, D. Kong, X. Li, Y. Wang, J. Sun, *ACS Nano* **2023**, *17*, 10844.

[21] Z. Wang, Z. Huang, H. Wang, W. Li, B. Wang, J. Xu, T. Xu, J. Zang, D. Kong, X. Li, H. Y. Yang, Y. Wang, *ACS Nano* **2022**, *16*, 9105.

[22] H. Wang, W. Bai, H. Wang, D. Kong, T. Xu, Z. Zhang, J. Zang, X. Wang, S. Zhang, Y. Tian, X. Li, C.-S. Lee, Y. Wang, *Energy Storage Mater.* **2023**, *55*, 631.

[23] D. Pan, H. Yang, Y. Liu, H. Wang, T. Xu, D. Kong, J. Yao, Y. Shi, X. Li, H. Y. Yang, Y. Wang, *Nanoscale* **2023**, *15*, 17482.

[24] H. Yang, H. Wang, W. Li, B. Tian, T. Xu, D. Kong, S. Huang, K. Liu, X. Li, H. Y. Yang, Y. Wang, *J. Mater. Chem. A* **2022**, *10*, 16842.

[25] W. Bai, H. Wang, D. H. Min, J. Miao, B. Li, T. Xu, D. Kong, X. Li, X. Yu, Y. Wang, H. S. Park, *Adv. Sci.* **2024**, *11*, 2404419.

[26] Y. Liu, H. Wang, D. Pan, J. Hou, J. Yao, D. Kong, T. Xu, Y. Shi, X. Li, H. Y. Yang, Y. Wang, Z. S. Wu, *Adv. Funct. Mater.* **2024**, *34*, 2405460.

[27] B. Huang, S. Sun, J. Wan, W. Zhang, S. Liu, J. Zhang, F. Yan, Y. Liu, J. Xu, F. Cheng, Y. Xu, Y. Lin, C. Fang, J. Han, Y. Huang, *Adv. Sci.* **2023**, *10*, 2206845.

[28] C. Bao, J. Wang, B. Wang, J. Sun, L. He, Z. Pan, Y. Jiang, D. Wang, X. Liu, S. X. Dou, J. Wang, *ACS Nano* **2022**, *16*, 17197.
